# Tetrazine‐Triggered Bioorthogonal Cleavage of *trans*‐Cyclooctene‐Caged Phenols Using a Minimal Self‐Immolative Linker Strategy**

**DOI:** 10.1002/cbic.202200363

**Published:** 2022-08-30

**Authors:** Patrick Keppel, Barbara Sohr, Walter Kuba, Marion Goldeck, Philipp Skrinjar, Jonathan C. T. Carlson, Hannes Mikula

**Affiliations:** ^1^ Institute of Applied Synthetic Chemistry TU Wien 1060 Vienna Austria; ^2^ Center for Anatomy and Cell Biology Medical University of Vienna 1090 Vienna Austria; ^3^ Center for Systems Biology & Department of Medicine Massachusetts General Hospital Harvard Medical School Boston, MA 02114 USA

**Keywords:** bioorthogonal chemistry, click chemistry, cycloaddition, elimination, prodrugs

## Abstract

Bond‐cleavage reactions triggered by bioorthogonal tetrazine ligation have emerged as strategies to chemically control the function of (bio)molecules and achieve activation of prodrugs in living systems. While most of these approaches make use of caged amines, current methods for the release of phenols are limited by unfavorable reaction kinetics or insufficient stability of the Tz‐responsive reactants. To address this issue, we have implemented a self‐immolative linker that enables the connection of cleavable *trans*‐cyclooctenes (TCO) and phenols via carbamate linkages. Based on detailed investigation of the reaction mechanism with several Tz, revealing up to 96 % elimination after 2 hours, we have developed a TCO‐caged prodrug with 750‐fold reduced cytotoxicity compared to the parent drug and achieved *in situ* activation upon Tz/TCO click‐to‐release.

## Graphical abstract

**Bioorthogonal activation** of caged phenols enables strategies to control the function of (bio)molecules in biological environments. While previous methods have been limited by slow click kinetics or insufficient stability, we show that introduction of a self‐immolative linker affords stable *trans*‐cyclooctene conjugates, as demonstrated by the design of a prodrug that upon bioorthogonal reaction with tetrazines leads to >200‐fold cytotoxicity.

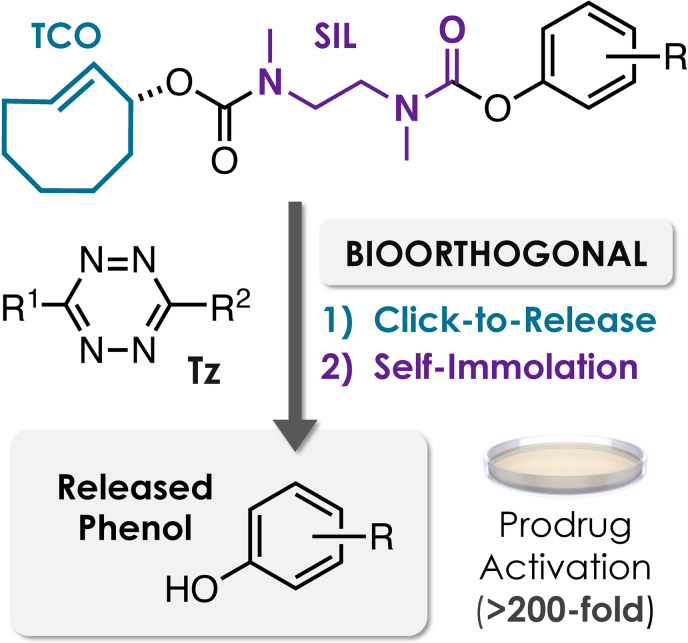

## Introduction

Biocompatible bond‐cleavage reactions have expanded the repertoire of bioorthogonal tools substantially, providing methods beyond chemical ligation to control the function of (bio)molecules in biological environments and living systems.^[1]^ Bioorthogonal elimination has been achieved by applying transition metals or click‐type reactions to trigger the cleavage of a linker and/or the release of a molecule.^[2]^ So far, these methods have mainly been applied to control (i) the cleavage of ligand‐drug conjugates and (ii) the activation of prodrugs, thereby facilitating the release of therapeutics.^[3]^ In addition, strategies to activate caged proteins^[4]^ (‘turn‐on’) and to cleave imaging probes^[5]^ (‘turn‐off’) have been designed, expanding the scope of bioorthogonal ON/OFF control.

**Figure 1:**
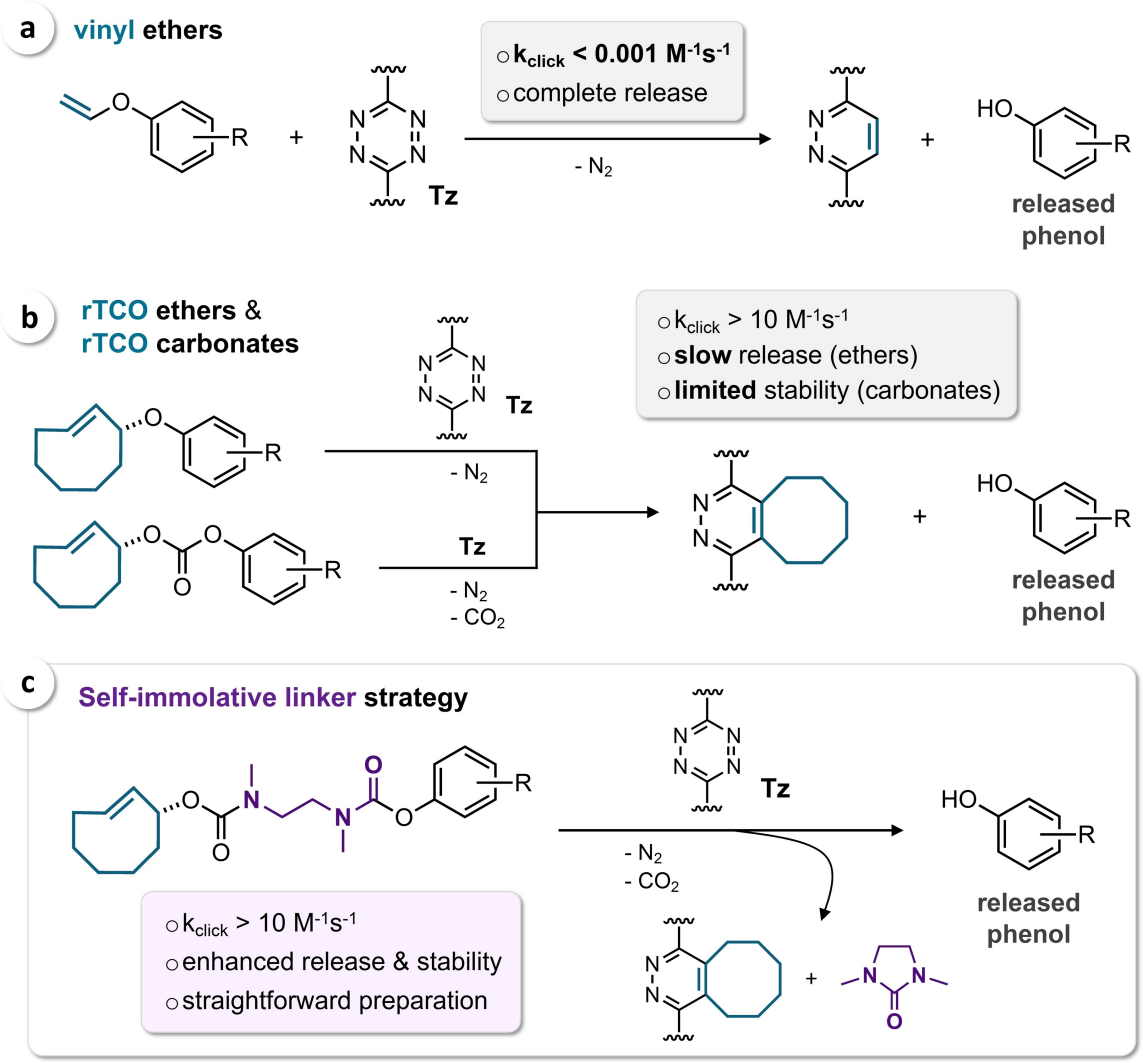
(a) Bioorthogonal cleavage of vinyl ethers via inverse electron demand Diels‐Alder reaction with tetrazines (Tz). (b) Tz‐triggered release of phenols from rTCO ethers and carbonates. (c) Incorporation of a minimal self‐immolative linker enables the design of rTCO‐caged phenols with enhanced stability that can be cleaved efficiently by reaction with Tz.

The bioorthogonal elimination of *trans*‐cyclooctenes (TCO) triggered by click reaction with 1,2,4,5‐tetrazines (Tz) has gained significant attention due to exceptional reaction kinetics and molecular versatility.^[6]^ As pioneered by Robillard *et al*., TCO modified with a carbamate moiety in an allylic position (release‐TCO, rTCO) undergoes a 1,4‐elimination upon inverse electron demand Diels‐Alder (IEDDA) reaction with Tz and subsequent post‐click tautomerization, ultimately leading to the click‐triggered release of an amine or amino‐functionalized molecule (*click‐to‐release*).^[7]^ To expand the scope of Tz as molecular scissors to the activation of caged alcohols and phenols, vinyl ethers have been introduced as bioorthogonal reactants (Figure 1a), representing a strategy for the design of a variety of prodrugs, considering that OH‐functionalized compounds account for a large number of FDA‐approved small‐molecule therapeutics.^[8]^ However, only limited second‐order rate constants (k_2_) of less than 0.001 M^−1^ s^−1^ have been observed for the IEDDA reaction of vinyl ethers and Tz.^[8]^ Versteegen *et al*. have developed rTCO‐ethers and rTCO‐carbonates that react substantially faster with Tz (k_2_>10 M^−1^ s^−1^), leading to click‐triggered release of alcohols and phenols (Figure 1b).^[9]^ However, in the case of rTCO‐ethers only slow elimination was observed, requiring >30 hours to obtain >80 % of the released alcohol or phenol. While click‐to‐release of phenolic rTCO‐carbonates was found to proceed significantly faster (80 % after approx. 15 hours), these compounds were reported to degrade rapidly under biological conditions (complete hydrolysis after 5 hours in 50 % mouse serum at 37 °C),^[9]^ impeding application of this strategy in the design of click‐activatable prodrugs. Further approaches include the use of a self‐immolative 4‐aminobenzyl alcohol linker between rTCO and the payload (release yields <40 %),^[10]^ and the Tz‐triggered cleavage of 3‐isocyanopropyl‐caged phenols (k_2_ <5 M^−1^ s^−1^).^[11]^

To facilitate efficient bioorthogonal cleavage of rTCO‐caged phenols while achieving high precursor stability, we envisioned the implementation of a self‐immolative linker (SIL) that enables the use of carbamate linkages instead of ethers and carbonates. Here, we show that *N*,*N’*‐dimethylethylenediamine (DMEDA) can be used as a minimal SIL to achieve bioorthogonal elimination of phenols (Figure 1c). Depending on the structure of the Tz and the cleavable TCO we observed up to 96 % release after 2 hours, while achieving high stability of the caged phenol under physiological conditions, as demonstrated by the design of a bioorthogonal prodrug with 750‐fold reduced cytotoxicity.

## Results and Discussion

DMEDA is commonly used as a self‐immolative linker or spacer in the design of cleavable and/or activatable compounds such as drug conjugates.^[12]^ Replacement of an *N*‐methyl substituent with other functionalities moreover allows further modification, as very recently applied for the development of an rTCO‐caged fluorogenic resorufin derivative.^[13]^

**Figure 2:**
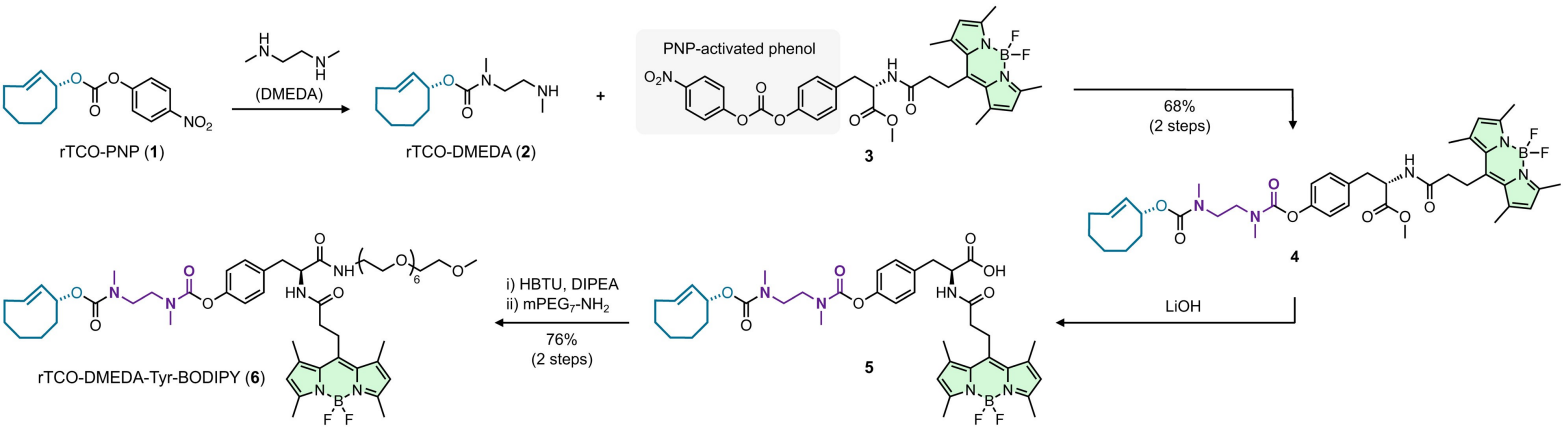
Chemical synthesis of the water‐soluble rTCO‐caged BODIPY‐labeled release probe **6**. The active carbonate rTCO‐PNP (**1**) was reacted with an excess of DMEDA to obtain compound **2**, which was subsequently coupled to the PNP‐activated tyrosine‐BODIPY conjugate **3**. Saponification of intermediate **4** and PEGylation of the resulting acid **5** via HBTU‐mediated amide coupling afforded rTCO‐DMEDA‐Tyr‐BODIPY (**6**).

For the detailed investigation of the reactions of rTCO‐DMEDA‐phenol conjugates with structurally different Tz we have first prepared a water‐soluble release probe labeled with a boron dipyrromethene (BODIPY) fluorophore that allows detection and relative quantification of all intermediates and products by HPLC using its characteristic absorption at a wavelength of 500 nm.^[14]^ The active *p*‐nitrophenyl (PNP) carbonate rTCO‐PNP (**1**) was treated with an excess of DMEDA and (following evaporation to afford crude intermediate **2**) reacted with the PNP‐activated tyrosine‐BODIPY conjugate **3** to obtain the rTCO‐DMEDA‐caged compound **4** in an overall yield of 68 %. Saponification and subsequent PEGylation of the intermediate acid **5** via HBTU‐mediated amide coupling yielded rTCO‐DMEDA‐Tyr‐BODIPY (**6**) with a yield of 76 % over 2 steps (Figure 2).

**Figure 3:**
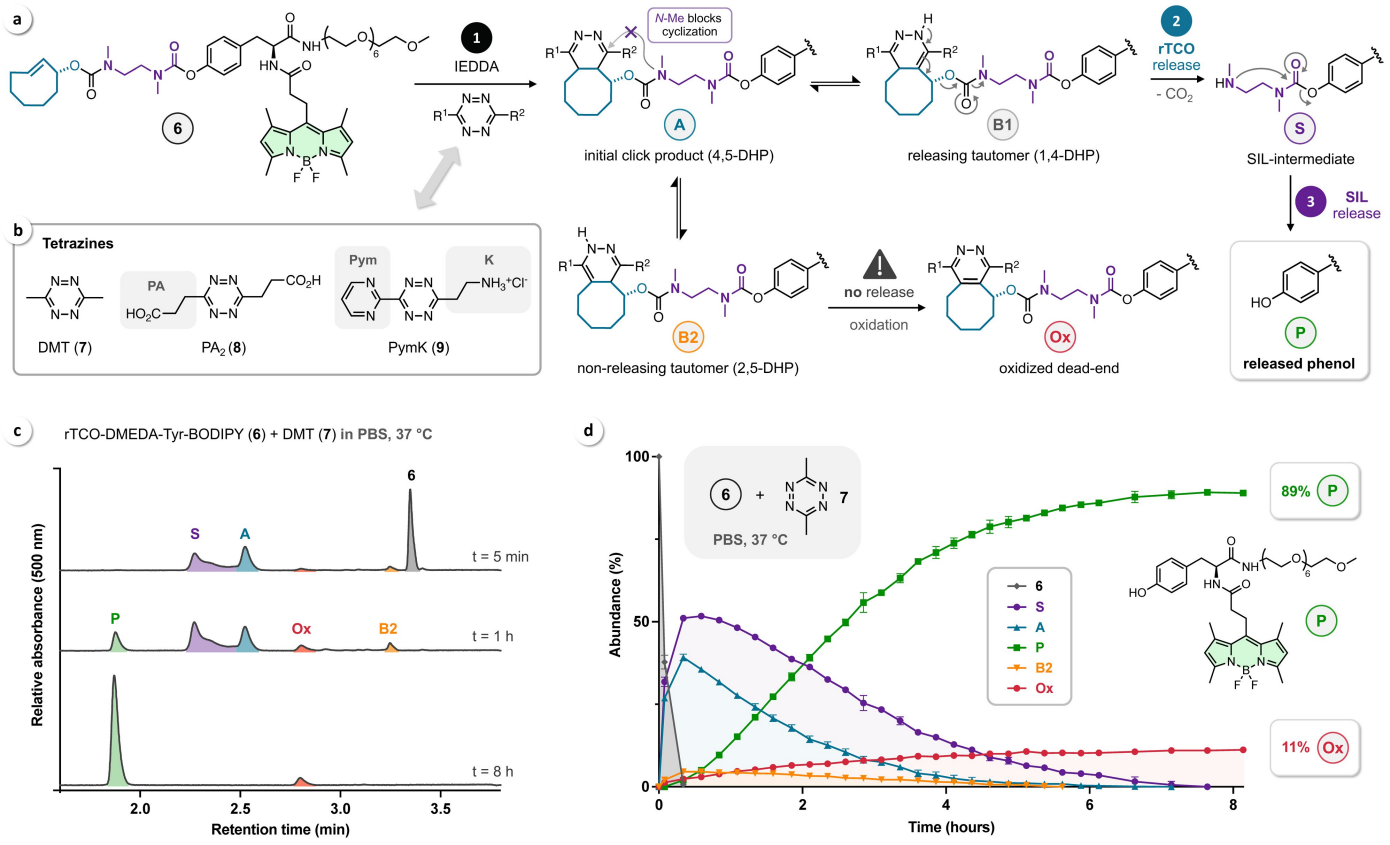
(a) Mechanism of IEDDA‐triggered click‐to‐release of probe **6**. (b) Chemical structures of selected Tz. (c) Serial chromatograms (buffered HPLC conditions, ammonium formate, 2.5 mM, pH 8.4) for the reaction of **6** (50 μM) and DMT (**7**, 100 μM) in phosphate‐buffered saline (PBS, 10 mM, pH 7.4) at 37 °C, using the characteristic BODIPY absorption at 500 nm for the detection and relative quantification of all reactants, intermediates, and reaction products. (d) Complete reaction profile (HPLC, n=3) for the click‐to‐release of **6** (50 μM) triggered by IEDDA‐ligation with DMT (**7**, 100 μM).

Click‐to‐release of **6** triggered by reaction with Tz was then studied by HPLC‐MS. According to the current mechanistic understanding,^[14]^ IEDDA ligation leads to formation of an initial 4,5‐dihydropyridazine (4,5‐DHP) click product **A** that can tautomerize to the releasing 1,4‐DHP isomer **B1** and the non‐releasing 2,5‐DHP isomer **B2** (Figure 3a). The *N*‐methyl group of the DMEDA linker blocks undesired intramolecular cyclization of **A**, thereby preventing the formation of a tricyclic dead‐end product that, as we have previously shown,^[14]^ could otherwise severely impact the outcome of the bioorthogonal bond‐cleavage process. rTCO‐release via 1,4‐elimination of **B1** yields the SIL‐intermediate **S** that upon cyclization of the DMEDA‐carbamate reacts to the uncaged phenol **P**. Depending on the chemical structure of the Tz, **B2** is in equilibrium with **A**, but can oxidize to the non‐releasing pyridazine **Ox** (Figure 3a). Based on previous reports, we have selected 3,6‐dimethyl‐1,2,4,5‐tetrazine (DMT, **7**),[^7^, ^14^] the diacid PA_2_ (**8**),^[14]^ and the ammonium‐functionalized tetrazine PymK (**9**)^[15]^ to investigate the IEDDA‐triggered click‐to‐release of **6** (Figure 3b). While second‐order rate constants (k_2_) of <100 M^−1^s^−1^ have been reported for the click reaction of rTCO with DMT (**7**) and PA_2_ (**8**), pyrimidyl‐alkyl‐Tz such as **9** are known to be substantially more reactive, reaching k_2_ of >1000 M^−1^ s^−1^ (see Supporting Information for k_2_ determined in PBS at 37 °C).[^5^, ^14^]

We have previously reported on the profound pH sensitivity of Tz/TCO‐release and its impact on HPLC analysis, with routinely applied acidic conditions (e. g., formic acid) leading to ‘*pseudo‐release*’ that impedes accurate reaction monitoring.^[14]^ Thus, buffered HPLC conditions (ammonium formate, 2.5 mM, pH 8.4) were used to investigate the reactions of **6** with Tz **7** and **8** in PBS at 37 °C. Despite a tailing peak for the SIL‐intermediate **S**, all reactants, tautomers, and products could be separated and identified (Figure 3c), allowing monitoring of the post‐click reaction network by serial HPLC‐MS measurements. We observed the formation of the initial click product **A** (at early time points synchronously to the consumption of **6**), the non‐releasing tautomer **B2**, and the SIL‐intermediate **S** (as the product of TCO‐release) that upon self‐immolation afforded phenol **P** with an overall yield of 89 % after a reaction time of approx. 8 hours. In agreement with our previous findings,^[14]^ no detectable concentration of **B1** was found due to rapid 1,4‐elimination of this labile intermediate. The respective pyridazine dead‐end **Ox** was identified as the only non‐releasing byproduct (11 %, Figure 3d).

**Figure 4:**
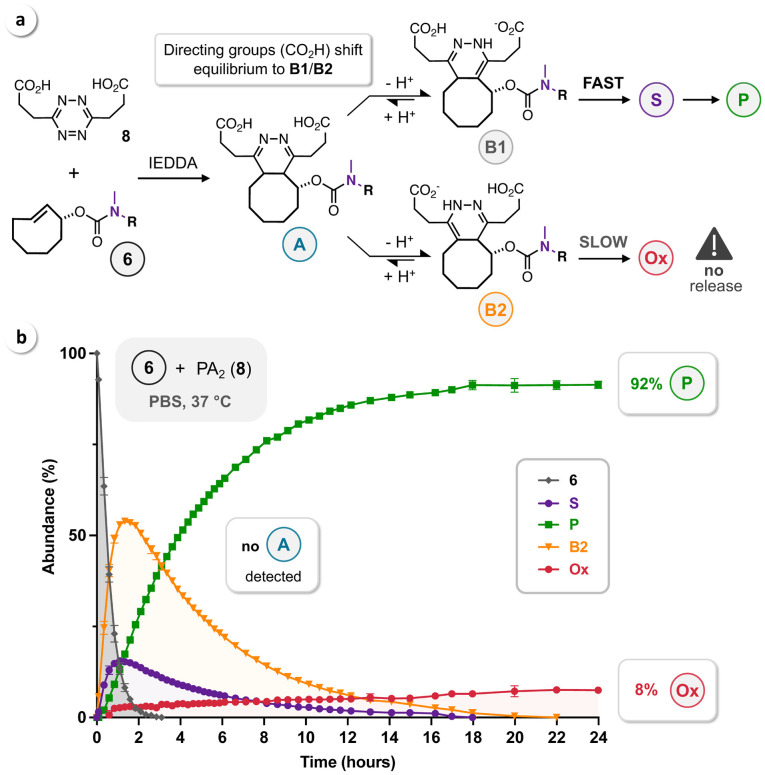
Click‐to‐release of rTCO‐DMEDA‐Tyr‐BODIPY (**6**) by reaction with the Tz‐diacid PA_2_ (**8**). (a) The directing CO_2_H groups of PA_2_ enhance tautomerization of **A** and thereby shift the equilibrium to **B1**/**B2**. (b) HPLC monitoring (n=3) of the reaction of **6** (50 μM) and **8** (100 μM) in PBS at 37 °C.

The diacid PA_2_ (**8**) is the only Tz reported so far that allows efficient release (>90 %) of primary amines.^[14]^ The two carboxylic acid functionalities direct post‐click tautomerization of **A** and shift the equilibrium to **B1**/**B2**, as shown for the reaction of **6** and **8** (PBS, 37 °C) in Figure 4a. Serial HPLC measurements confirmed this effect as the click product **A** was not detectable. Enhanced tautomerization leads to formation of instantaneously releasing **B1** and slowly oxidizing **B2**, which can still tautomerize to **B1** (via **A**), thereby enabling efficient release. In agreement with these mechanistic considerations, we observed 92 % release of the uncaged phenol **P** and 8 % of the respective oxidized dead‐end (Figure 4b). However, due to competition between the two directing CO_2_H groups,^[14]^ the overall release is significantly slower (approx. 20 hours to reach the endpoint) than observed for the reaction of **6** and DMT (**7**) (cf. Figure 3).

**Figure 5:**
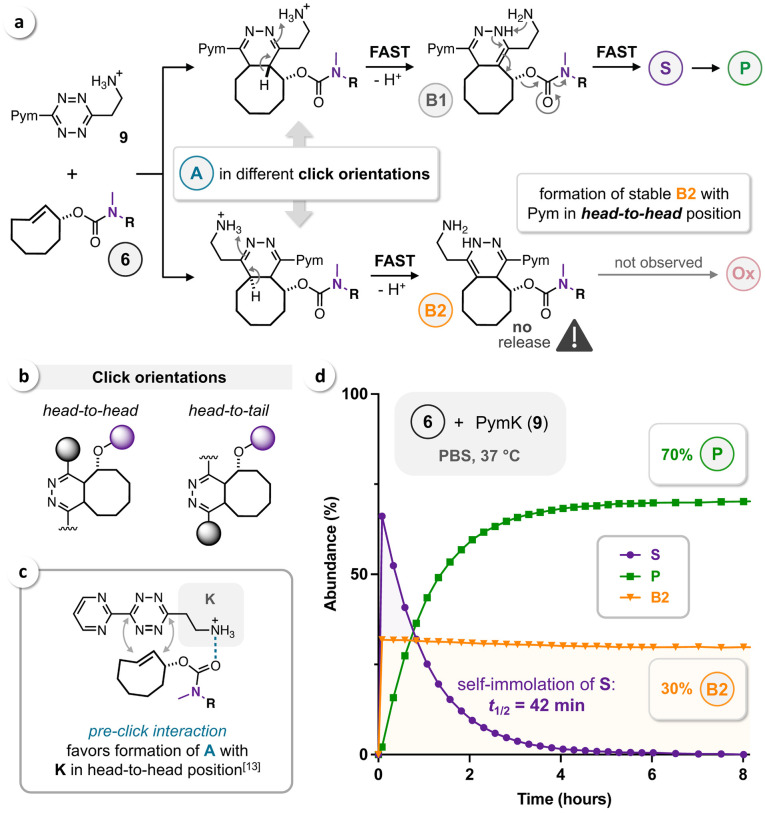
(a) Depending on the click orientation upon IEDDA ligation of **6** and PymK (**9**), the directing NH_3_^+^ group leads to fast TCO‐release or formation of a stable **B2** tautomer. (b) Click orientations: *head‐to‐head* vs. *head‐to‐tail*. (c) Pre‐click interaction of Tz‐NH_3_^+^ with the TCO‐carbamate moiety. (d) HPLC monitoring (n=3) of the reaction of **6** (50 μM) and **9** (100 μM) in PBS at 37 °C.

In contrast to the symmetric tetrazines **7** and **8**, IEDDA ligation of PymK (**9**) and rTCO leads to formation of two initial click products (Figure 5a). Depending on the click orientation, the NH_3_^+^‐directing group (K) is either on the same (*head‐to‐head*) or the opposite side (*head‐to‐tail*) of the leaving group in allylic position of the TCO (Figure 5b). Similar to unsymmetric Tz acids, as we have recently reported,^[5]^ NH_3_^+^ in head‐to‐head position accelerates TCO‐release via **B1**, while the head‐to‐tail orientation leads to irreversible formation of a stable and therefore non‐releasing **B2** (Figure 5a). Ammonium‐functionalized Tz were first described by van Kasteren *et al*.,^[15]^ who moreover discovered a pre‐click interaction of the NH_3_^+^ moiety with the TCO‐carbamate (Figure 5c). As a result, click in head‐to‐head orientation is favored, thereby improving the efficiency of the overall bond‐cleavage process. In addition, the release step upon click with ammonium‐functionalized Tz was reported to be instantaneous (when NH_3_^+^ is in head‐to‐head position)[^5^, ^16^] and independent from pH.^[15]^ We exploited that knowledge by using acidic HPLC conditions to achieve better separation of the intermediates (all bearing an NH_3_^+^ moiety) formed upon reaction of **6** with PymK (**9**) without provoking any pseudo‐release (for details see the Supporting Information). As expected, we observed immediate post‐click tautomerization (no **A** detected) and 1,4‐elimination (no **B1** detected) leading to formation of the SIL‐intermediate **S** and stable **B2** in a ratio of 7/3, which confirms favored click orientation with NH_3_^+^ in head‐to‐head position (Figure 5d). The uncaged phenol was obtained in a yield of 70 % after a total reaction time of approx. 8 hours (65 % after 3 hours). Due to the instantaneous TCO‐release, the self‐immolation of **S** could be fitted to a one‐phase exponential decay (R^2^=0.9998), revealing a first‐order rate constant (k_1_) of 1.0 h^−1^ for the cyclization of the DMEDA‐carbamate and a respective half‐life (*t*_1/2_) of 42 min (Figure 5d), which is similar to the *t*_1/2_ previously reported for a DMEDA‐carbamate of 4‐hydroxyanisole (36 min at pH 7.4 and 37 °C).^[17]^

**Figure 6:**
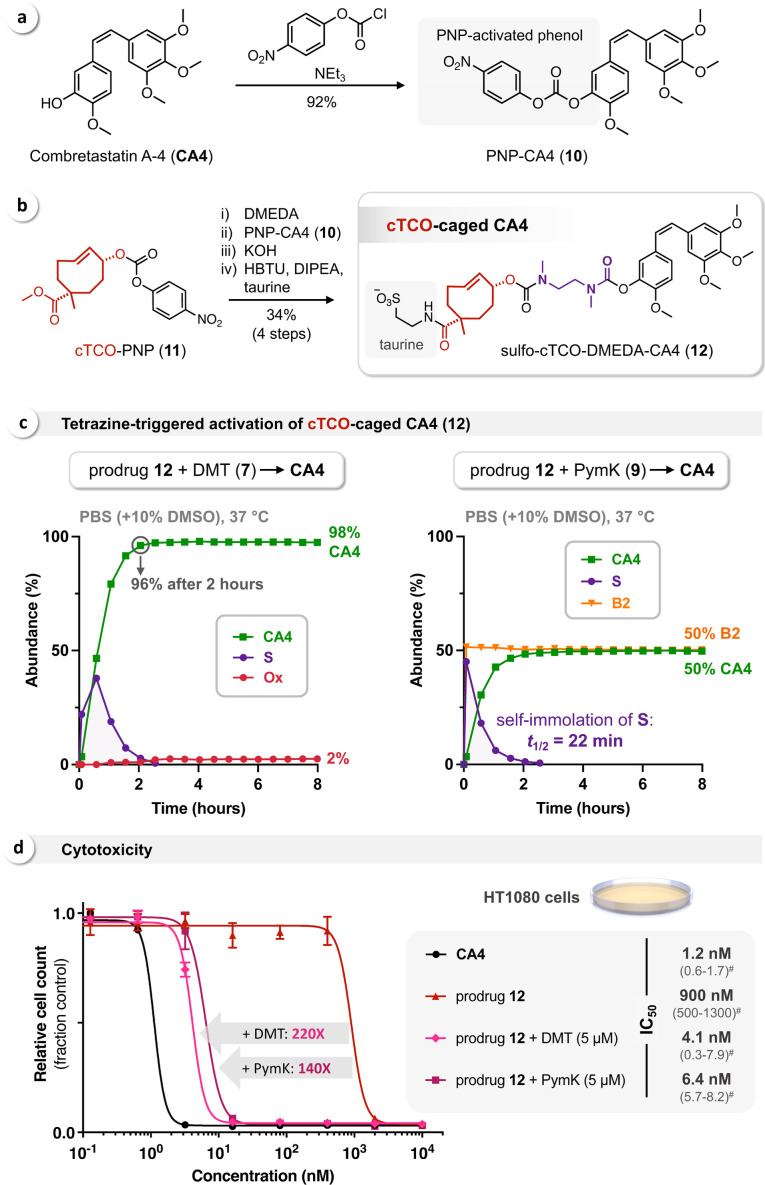
(a) Chemical structure of combretastatin A‐4 (**CA4**) and PNP‐activation of the free phenolic OH‐group. (b) Synthesis of the cTCO‐DMEDA‐caged prodrug **12**. (c) Monitoring (via HPLC‐MS) of the bioorthogonal activation of **12** (50 μM) triggered by click‐to‐release reaction with DMT (**7**, left) and PymK (**9**, right) at a concentration of 100 μM. (d) Cell viability assays using HT1080 fibrosarcoma cancer cells show a 750‐fold increased IC_50_ of **12** (900 nM) compared to **CA4** (1.2 nM), also demonstrating high stability of the bis(carbamate)‐linked prodrug. Cytotoxicity was restored by *in situ* reaction with DMT (**7**, 220‐fold) and PymK (**9**, 140‐fold), confirming release of **CA4** via Tz‐triggered bioorthogonal bond‐cleavage (^#^IC_50_ 95 % confidence intervals in nM).

To demonstrate application of the Tz/TCO‐DMEDA strategy for the bioorthogonal release of phenols, we designed a prodrug of the anti‐mitotic and anti‐angiogenic agent combretastatin A‐4 (**CA4**, Figure 6a).^[18]^ The unique yet simple chemical structure and the high potency of **CA4** have motivated the development of several therapeutic strategies, in particular by applying prodrugs such as CA4‐phosphate (CA‐4P, fosbretabulin) to overcome the poor water‐solubility of the parent drug.^[19]^

Straightforward modification of **CA4** was enabled by PNP‐activation of the free phenolic OH‐group yielding PNP‐CA4 (**10**) (Figure 6a). To facilitate sufficient water‐solubility we used cTCO as a Tz‐responsive cleavable linker,[^3c^, ^14^] allowing incorporation of a sulfonate moiety (via the additional functional group of cTCO) to obtain the CA4‐prodrug **12** (Figure 6b). To incorporate the SIL, cTCO‐PNP (**11**) was first treated with an excess of DMEDA and then reacted with PNP‐CA4 (**10**). Saponification of the methyl ester followed by HBTU‐mediated coupling of the intermediate acid with 2‐aminoethanesulfonic acid (taurine) afforded sulfo‐cTCO‐DMEDA‐CA4 (**12**) in an overall yield of 34 % (Figure 6b).

The bioorthogonal activation of **12** by reaction with tetrazines **7** and **9** was monitored by HPLC‐MS as described above. The relative abundance of all intermediates and products was calculated based on the UV absorbance at 254 nm and confirmed via quantification of released **CA4** by external calibration (see Supporting Information). In agreement with data previously reported,^[14]^ we observed a significantly faster Tz‐triggered elimination of cTCO compared to rTCO (cf. Figure 3). Reacting prodrug **12** with DMT (**7**) resulted in 96 % release of **CA4** already after 2 hours, reaching an endpoint of 98 % **CA4** and 2 % of the respective oxidized dead‐end after a total reaction time of approx. 4 hours (Figure 6c, left). In contrast, IEDDA ligation of **12** and PymK (**9**) did not lead to favored formation of the initial click product with the NH_3_^+^‐substituent (K) in head‐to‐head position, as indicated by an overall **CA4** release of 50 %. Accordingly, 50 % of non‐releasing stable **B2** with K in head‐to‐tail position was detected (Figure 6c, right). Nevertheless, this experiment allowed analysis of the self‐immolation kinetics, revealing accelerated cyclization of the DMEDA‐carbamate of **CA4** with a half‐life of 22 min (k_1_=1.9 h^−1^, one‐phase exponential fitting, R^2^=0.9993), which we hypothesize is due to **CA4** being a better leaving group than the tyrosine‐BODIPY conjugate **P** (cf. Figure 3 and Figure 5).

Based on these results we investigated the cytotoxicity of prodrug **12** and its bioorthogonal activation by reaction with Tz in HT1080 fibrosarcoma cancer cells.^[20]^ In comparison to the parent drug **CA4**, a 750‐fold higher concentration of cTCO‐caged **12** (900 nM) was needed to reduce the cell count by 50 % (IC_50_) over the course of a 72 h treatment (Figure 6d), demonstrating both efficient caging and the high stability of the bis(carbamate)‐linked cTCO‐DMEDA‐prodrug under physiologically relevant conditions (for additional data on the stability of prodrug **12** in PBS and cell growth medium see Supporting Information). *In situ* reaction of **12** with DMT (**7**) and PymK (**9**) at a non‐toxic Tz concentration of 5 μM restored drug cytotoxicity via Tz‐triggered bioorthogonal release of **CA4**, shifting the IC_50_ from 900 nM to 4.1 nM (220‐fold) and 6.4 nM (140‐fold), respectively (Figure 6d). As a point of reference, the cTCO‐caged prodrug SQP33, currently being tested in phase 1 clinical trials,^[21]^ was reported to be 83‐fold less cytotoxic than its parent drug doxorubicin.^[3g]^ To confirm the mechanistic basis of cytotoxicity and anti‐mitotic effect upon click activation of **12**, differentially treated HT1080 cells were stained with SiR‐tubulin,^[22]^ a fluorescent probe that enables visualization of intact endogenous microtubules via fluorescence microscopy. While efficient staining of the cellular microtubule network was observed upon treatment with either **12** or DMT (**7**) alone, only very low signal was detected upon bioorthogonal release of **CA4** via *in situ* reaction of both compounds (see Supporting Information, Figure S1), matching the **CA4** control and its expected impact on tubulin polymerization.

## Conclusion

In summary, we show that DMEDA can be applied as a minimal self‐immolative linker for the design of bis(carbamate)‐linked TCO‐caged phenols that can be cleaved and thereby activated by bioorthogonal reaction with tetrazines. The mechanism of the overall bond‐cleavage process upon IEDDA‐ligation with selected symmetrical and unsymmetrical Tz was investigated in detail, with emphasis on Tz‐substituents modified with directing groups (CO_2_H, NH_3_^+^) and their effect on post‐click tautomerization and 1,4‐elimination. We achieved >95 % release of the phenolic drug **CA4** upon Tz‐triggered cleavage of prodrug **12** in PBS within 2 hours. Notably, we have observed a 750‐fold lower cytotoxicity of the prodrug compared to **CA4**, also demonstrating high stability of TCO‐DMEDA‐phenol conjugates. *In situ* reaction of **12** with Tz confirmed efficient bioorthogonal release of **CA4** resulting in a substantially (up to 220‐fold) increased cytotoxicity. Based on these results, we aim to combine TCO‐DMEDA‐caging with next‐level chemical tools for bioorthogonal bond‐cleavage to control post‐click tautomerization and elimination, thereby accelerating the Tz/TCO click reaction while achieving fast and complete release of phenolic drugs.

## Experimental Section

**General methods**: Preparative HPLC and flash chromatography were carried out on a Grace REVELERIS Prep purification system using a Kinetex 5 μm C18 100 Å, AXIA Packed LC Column 100×30.0 mm (Phenomenex) for preparative RP‐HPLC or a Luna 10 μm Silica (2) 100 Å, LC Column 250×21.2 mm (Phenomenex) for preparative NP‐HPLC purifications. Silica gel 60 (40‐63 μm) was purchased from Merck. HPLC‐MS (LCMS) analysis was performed on a Nexera X2 system (Shimadzu) comprised of LC‐30AD pumps, a SIL‐30AC autosampler, a CTO‐20AC column oven, and a DGU‐20 A_5/3_ degasser module. Detection was done using an SPD−M20 A photo diode array, an RF‐20Axs fluorescence detector, an ELS‐2041 detector (JASCO) and an LCMS‐2020 mass spectrometer (ESI). All separations were performed using a Waters XSelect CSH C18 2.5 μm (3.0×50 mm) column XP at 40 °C and a flowrate of 1.7 mL/min. Used gradients (unless otherwise noted, see Supporting Information): Acidic HPLC conditions (acetonitrile/0.1 % formic acid): 0 min, 5 % – 0.15 min, 5 % – 2.20 min, 98 % – 2.50 min, 98 %; buffered HPLC conditions (acetonitrile/2.5 mM ammonium formate buffer, pH 8.4): 0 min, 5 % – 0.15 min, 5 % – 2.2 min, 98 % – 2.5 min; 98 %. ^1^H and ^13^C NMR spectra were recorded on a Bruker Ascend 600 MHz spectrometer at 20 °C. Chemical shifts (δ) are reported in ppm relative to tetramethylsilane and calibrated using solvent residual peaks. HRMS analysis of aqueous or acetonitrile solutions of the compounds (sample concentration: 10 ppm) was carried out on an Agilent 6545 Q‐TOF mass spectrometer equipped with an Agilent Dual AJS ESI source. The mass spectrometer was connected to a liquid chromatography system comprised of an Agilent G7167B multi sampler, an Agilent G7120 A binary pump with degasser and an Agilent G7116B oven (Agilent). A SecurityGuard Cartridge (Phenomenex) was used as a stationary phase. Data evaluation was performed using Agilent MassHunter Workstation Qualitative Analysis 10.0. Stopped‐flow measurements were performed using an SX20‐LED stopped‐flow spectrophotometer (Applied Photophysics) equipped with a 535 nm LED to monitor the characteristic tetrazine visible light absorbance (520‐540 nm).

**Synthesis**: Detailed experimental procedures for the synthesis of compounds **2**–**6**, **10**–**12** and further intermediates are provided in the Supporting Information, including compound characterization data (NMR, MS, HPLC).

**Click‐to‐release monitoring (HPLC)**: Click‐to‐release reactions were performed in HPLC vials at 37 °C using the temperature‐controlled HPLC autosampler (see General Methods). For acidic HPLC conditions, the aqueous solvent was prepared by addition of 2.5 mL of formic acid to 2.5 L of HPLC‐water to yield a final concentration of 0.1 % formic acid. For buffered HPLC conditions, the aqueous solvent was prepared by addition of 625 μL of 10 M ammonium formate (BioUltra, Sigma‐Aldrich) to 2.5 L of HPLC‐grade water followed by adjusting the pH to 8.4 by addition of 25 % aqueous ammonia (for HPLC, LiChropur, Merck). Since its pH declines over time, this volatile buffer was freshly prepared each day. HPLC‐grade acetonitrile was used without any additives. Stock solutions of rTCO‐DMEDA‐Tyr‐BODIPY (**6**) and sulfo‐cTCO‐DMEDA‐CA4 (**12**) were prepared at a concentration of 20 mM in DMSO. Tetrazine stock solutions of DMT (**7**), PA_2_ (**8**) and PymK (**9**) were prepared at a concentration of 10 mM in DMSO.

rTCO‐DMEDA‐Tyr‐BODIPY (**6**): The stock solution of **6** was diluted with PBS to a concentration of 100 μM in an LCMS vial (2.5 μL 20 mM TCO stock, 497.5 μL PBS). Tetrazine stock solutions were diluted with PBS to a concentration of 200 μM in an LCMS vial (2.5 μL Tz stock, 122.5 μL PBS), and the click‐to‐release reaction was initiated by addition of the diluted TCO solution (125 μL) to obtain starting concentrations of 50 μM TCO and 100 μM Tz. The samples were immediately incubated at 37 °C in the autosampler and subjected to serial HPLC analysis. All measurements were conducted in triplicates.

sulfo‐cTCO‐DMEDA‐CA4 (**12**): The stock solution of **12** was diluted with DMSO (30 μL TCO stock, 30 μL DMSO) to give a 10 mM stock solution. Tz stock solution (10 μL) was added to PBS (985 μL, containing 8.6 % DMSO), and the click‐to‐release reaction was initiated by addition of the stock solution of **12** (5.24 μL) to obtain starting concentrations of 50 μM TCO and 100 μM Tz (in 10 % DMSO/PBS). The samples were immediately incubated at 37 °C in the autosampler and subjected to serial HPLC analysis. All measurements were conducted in triplicates.

Further details on the determination of the exact TCO concentration of stock solutions, and analytical HPLC analyses are provided in the Supporting Information, including selected chromatograms and MS data.

**Click kinetics (stopped‐flow spectrophotometry)**: An approx. 20 mM stock solution of **rTCO‐PEG_4_** ^[14]^ in DMSO was prepared. The exact concentration was determined by absorbance titration with DMT (**7**) (extinction coefficient 510 M^−1^ cm^−1^ at 520 nm), quantifying the decrease in tetrazine absorbance upon reaction with TCO. The initial DMSO stock was diluted with PBS to prepare solutions for stopped‐flow analysis at a final TCO concentration of 1 mM. Stock solutions (20 mM) of DMT (**7**), PA_2_ (**8**), and PymK (**9**) in DMSO were prepared. Serial dilution into PBS gave solutions for stopped‐flow analysis at a Tz concentration of 100 μM. The reagent syringes of the stopped‐flow spectrophotometer (see General Methods) were loaded with tetrazine and TCO solutions and the instrument was primed. Stopped‐flow measurements were done in sextuplicate for each Tz. Reactions were conducted at 37 °C and recorded automatically at the time of acquisition. Data sets were analyzed by fitting an exponential decay using Prism 6 (GraphPad) to calculate the observed pseudo‐first‐order rate constants that were converted into second‐order rate constants (Table S1, see Supporting Information) by dividing through the TCO concentration.

**Cell viability assays**: HT1080 human fibrosarcoma cells (ATCC) were cultivated in EMEM (Minimum Essential Medium Eagle, with Earle′s salts, l‐glutamine and sodium bicarbonate; Sigma Aldrich) supplemented with 10 % fetal bovine serum and 1 % antibiotic/antimycotic solution (100X, Sigma‐Aldrich) at 37 °C and 5 % CO_2_. HT1080 cells were seeded into 96‐well plates (triplicates for each group) at 10.000 cells per well and allowed to grow overnight. The medium was removed and a dilution series of sulfo‐cTCO‐DMEDA‐CA4 (**12**) or the parent drug **CA4** in growth medium (10 μM, 2 μM, 0.4 μM, 0.08 μM, 0.016 μM, 0.0032 μM, 0.00064 μM, 0.000128 μM, 0.0000256 μM) was added to the cells (0.1 % final DMSO concentration). For release experiments the same concentrations of **12** were used, while a stock solution of DMT (**7**) or PymK (**9**) was added to obtain final concentration of 5 μM of the respective tetrazine. Incubation was carried out for 72 h. Cell viability was assessed by replacing the medium with 100 μL of PrestoBlue solution (Invitrogen, 1 : 9 in growth medium) followed by incubation for 30 minutes at 37 °C. Read‐out of the fluorescence signal was carried out using a PerkinElmer EnSpire Multimode Plate Reader and data processing was done in GraphPad Prism. Following the same procedure, cells were treated with DMT (**7**), PymK (**9**), or 1,3‐dimethylimidazolidin‐2‐one (=byproduct of the self‐immolation process) with concentrations of up to 10 μM, revealing no significant effect on cell viability.

**Prodrug stability and cell imaging**: Additional data and experimental details regarding (i) the stability of prodrug **12** in PBS and cell growth medium and (ii) confirmation of the mechanistic basis of cytotoxicity upon click activation of **12** via fluorescence microscopy after microtubules staining are included in the Supporting Information.

## Conflict of interest

The authors declare no conflict of interest.

1

## Supporting information

As a service to our authors and readers, this journal provides supporting information supplied by the authors. Such materials are peer reviewed and may be re‐organized for online delivery, but are not copy‐edited or typeset. Technical support issues arising from supporting information (other than missing files) should be addressed to the authors.

Supporting Information

## Data Availability

The data that support the findings of this study are available from the corresponding author upon reasonable request.
