# Supplementary material for: Tetrazine‐Triggered Bioorthogonal Cleavage of trans‐Cyclooctene‐Caged Phenols Using a Minimal Self‐Immolative Linker Strategy
Source: Chembiochem. 2022 Aug 30;23(20):e202200363. doi: 10.1002/cbic.202200363 (PMC9804162; doi:10.1002/cbic.202200363)
Supplement: Supplementary file 1 — Supporting Information [file CBIC-23-0-s001.pdf]

ChemBioChem

Supporting Information

Tetrazine-Triggered Bioorthogonal Cleavage of *trans*-Cyclooctene-Caged Phenols Using a Minimal Self-Immolative Linker Strategy**

Patrick Keppel[+], Barbara Sohr[+], Walter Kuba, Marion Goldeck, Philipp Skrinjar, Jonathan C. T. Carlson, and Hannes Mikula*

Table of Contents

1 General Methods S2

2 Synthesis S3

3 Release Experiments S7

4 Click Kinetics S15

5 Cell Viability Assays S15

6 Cell Imaging S16

7 Prodrug Stability S17

8 NMR Spectra, Chromatograms and MS Data S18

9 References S30

1 General Methods

Unless otherwise noted, reactions were carried out under an atmosphere of argon in air-dried glassware with magnetic stirring. Air- and/or moisture-sensitive liquids were transferred via syringe. All reagents were purchased from commercial suppliers and used without further purification. Combretastatin A-4 (**CA4**) was obtained from BLD Pharmatech. Amberlite IR120 hydrogen form ion-exchange resin was purchased from Sigma Aldrich and washed prior use. Dichloromethane, ethyl acetate and methanol were obtained from Donau Chemie AG. Dichloromethane was dried using PURESOLV-columns (Innovative Technology). Dry DMSO and DMF were obtained from ACROS Organics and stored under argon. Organic solvents used for preparative HPLC were purchased from VWR (acetonitrile) and Sigma Aldrich (hexane). HPLC-grade water was obtained by using a Purelab Chorus 1 water purification system (ELGA).

Thin layer chromatography was performed using TLC alumina plates (Merck, silica gel 60, fluorescence indicator F254). Visualization of the spots was achieved either by UV irradiation (254 or 365 nm) or by heat staining with ceric ammonium molybdate in ethanol/sulfuric acid.

Preparative HPLC and flash chromatography were carried out on a Grace REVELERIS Prep purification system using a Kinetex 5 µm C18 100 Å, AXIA Packed LC Column 100 x 30.0 mm (Phenomenex) for preparative RP-HPLC or a Luna 10 µm Silica (2) 100 Å, LC Column 250 x 21.2 mm (Phenomenex) for preparative NP-HPLC purifications. Silica gel 60 (40-63 µm) was purchased from Merck.

HPLC-MS (LCMS) analysis was performed on a Nexera X2 system (Shimadzu) comprised of LC-30AD pumps, a SIL-30AC autosampler, a CTO-20AC column oven, and a DGU-20A$_{5/3}$ degasser module. Detection was done using an SPD-M20A photo diode array, an RF-20Axs fluorescence detector, an ELS-2041 evaporative light scattering detector (JASCO) and an LCMS-2020 mass spectrometer (ESI/APCI). If not stated otherwise, all separations were performed using a Waters XSelect CSH™ C18 2.5 µm (3.0 x 50 mm) column XP at 40 °C and a flowrate of 1.7 mL/min with 0.1% aqueous formic acid or ammonium formate buffer (2.5 mM, pH 8.4) and acetonitrile (gradient elution). Acidic HPLC conditions (acetonitrile/0.1% formic acid) 0 min: 5%, 0.15 min: 5%, 2.20 min: 98%, 2.50 min: 98%; Buffered HPLC conditions (acetonitrile/2.5 mM ammonium formate buffer, pH 8.4) 0 min: 5%, 0.15 min: 5%, 2.20 min: 98%, 2.50 min: 98%. See section 3 for further details on buffer preparation.

^{1}H and ^{13}C NMR spectra were recorded on a Bruker Ascend 600 MHz spectrometer at 20 °C. Chemical shifts (δ) are reported in ppm relative to tetramethylsilane and calibrated using solvent residual peaks. Data is shown as follows: Chemical shift, multiplicity (s = singlet, d = doublet, t = triplet, q = quartet, quint = quintet, m = multiplet, br = broad signal), coupling constants (*J*, Hz) and integration.

HRMS analysis of aqueous or acetonitrile solutions of the compounds (sample concentration: 10 ppm) was carried out on an Agilent 6545 Q-TOF mass spectrometer equipped with an Agilent Dual AJS ESI source. The mass spectrometer was connected to a liquid chromatography system comprised of an Agilent G7167B multi sampler, an Agilent G7120A binary pump with degasser and an Agilent G7116B oven (Agilent Technologies, Palo Alto, CA, USA). A SecurityGuard Cartridge (Phenomenex) was used as a stationary phase. Data evaluation was performed using Agilent MassHunter Workstation Qualitative Analysis 10.0. Identification was based on peaks obtained from extracted ion chromatograms (extraction width ± 20 ppm).

2 Synthesis

rTCO-caged BODIPY-labeled released probe

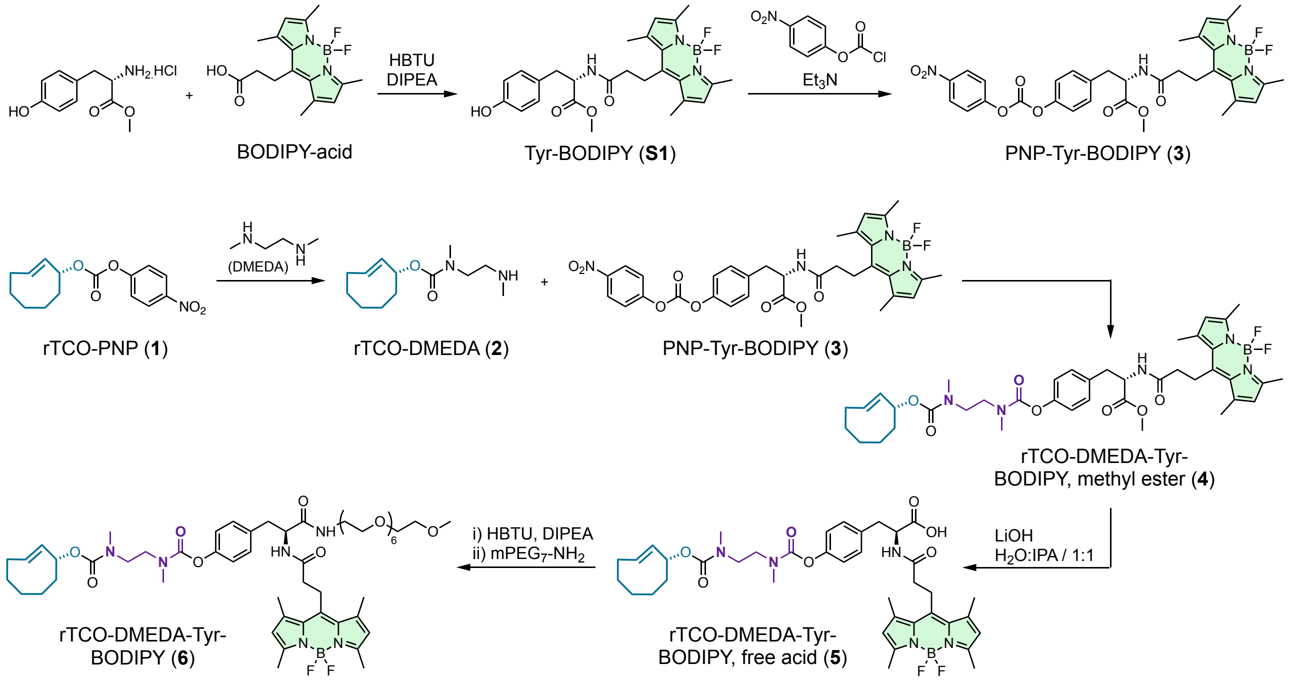

Tyr-BODIPY (S1)

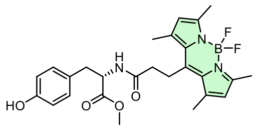

To a solution of BODIPY-acid[1] (20 mg, 62.5 µmol) in DMSO (4 mL) were added L-tyrosine methyl ester hydrochloride (40.5 mg, 174.9 µmol), HBTU (35.5 mg, 93.7 µmol) and DIPEA (53.1 µL, 40.3 mg, 312 µmol). LCMS analysis indicated complete conversion to the desired product after stirring for 1 h at room temperature (rt). Purification by preparative RP-HPLC (H_2O/MeCN gradient elution, 0.1% formic acid) afforded **S1** as an orange solid (28.0 mg, 90%). ^{1}H NMR (600 MHz, CD_2Cl_2) δ 6.91 (d, *J* = 8.4 Hz, 2H), 6.69 (d, *J* = 8.5 Hz, 2H), 6.13 – 6.01 (m, 4H), 4.85 – 4.79 (m, 1H), 3.72 (s, 3H), 3.30 – 3.17 (m, 2H), 3.08 (dd, *J* = 14.1, 5.6 Hz, 1H), 2.93 (dd, *J* = 14.1, 6.5 Hz, 1H), 2.48 – 2.33 (m, 14H); ^{13}C NMR (151 MHz, CD_2Cl_2) δ 172.36, 170.63, 155.70, 154.82, 144.69, 141.22, 131.58, 130.69, 127.86, 122.21, 115.80, 53.78, 52.76, 37.36, 37.25, 24.06, 16.61, 14.59; HRMS $[M+Na]^+$ calcd. 520.2189 for $C_{26}H_{30}BF_2N_3O_4Na^+$, found 520.2210.

PNP-Tyr-BODIPY (3)

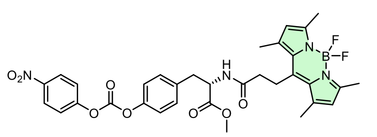

To a solution of **S1** (28.0 mg, 56.3 µmol) in CH_2Cl_2 (4 mL) was added a solution of 4-nitrophenyl chloroformate (PNP chloroformate) (12.5 mg, 61.9 µmol) in CH_2Cl_2 (1 mL) and Et_3N (18.8 µL, 13.7 mg, 135.1 µmol). The reaction mixture was heated to reflux for 1 h after which TLC indicated full consumption of the starting material. Purification by preparative HPLC (hexane/EtOAc gradient elution) afforded **3** as an orange solid (34.5 mg, 93%). ^{1}H NMR (600 MHz, CD_2Cl_2) δ 8.33 – 8.27 (m, 2H), 7.51 – 7.46 (m, 2H), 7.23 – 7.19 (m, 2H), 7.19 – 7.15 (m, 2H), 6.08 (s, 2H), 6.04 (d, *J* = 7.8 Hz, 1H), 4.88 (dt, *J* = 7.8, 6.1 Hz, 1H), 3.73 (s, 3H), 3.31 – 3.18 (m, 3H), 3.07 (dd, *J* = 14.0, 6.4 Hz, 1H), 2.51 – 2.39 (m, 14H); ^{13}C NMR (151 MHz, CD_2Cl_2) δ 172.00, 170.27, 155.67, 154.80, 151.50, 150.22, 146.09, 144.82, 141.19, 135.25, 131.60, 130.92, 125.75, 122.25, 122.18, 121.30, 53.51, 52.81, 37.57, 37.35, 24.11, 16.63, 14.59; HRMS $[M+Na]^+$ calcd. 685.2252 for $C_{33}H_{33}BF_2N_4O_8Na^+$, found 685.2280.

rTCO-DMEDA-Tyr-BODIPY, methyl ester (4)

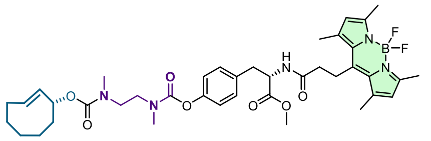

A solution of rTCO-PNP (**1**)[2] (axial isomer, 20.2 mg, 69.5 µmol) in CH_2Cl_2 (4 mL) was added via syringe pump (30 µL/min) to a solution of *N*,*N'*-dimethylethylenediamine (DMEDA) (74.9 µL, 61.3 mg, 695 µmol) in CH_2Cl_2 (1.2 mL) and DMF (0.4 mL) at 0 °C. Upon complete addition after 2 h, stirring at 0 °C was continued for 1 h. Excess of DMEDA was then removed under high vacuum (3 h) to obtain crude rTCO-DMEDA (**2**), which was then redissolved in CH_2Cl_2 (860 µL) and added to a solution of **3** (30.7 mg, 46.3 µmol) in DMF (430 µL) at rt. LCMS indicated complete conversion after a reaction time of 1 h. Purification by preparative HPLC (hexane/EtOAc gradient elution) afforded **4** as an orange solid (24.0 mg, 68%). ^{1}H NMR (600 MHz, CD_2Cl_2, mixture of rotamers) δ 7.10 – 7.04 (m, 2H), 7.03 – 6.96 (m, 2H), 6.09 (s, 2H), 6.06 (s, 1H), 5.85 – 5.71 (m, 1H), 5.58 – 5.46 (m, 1H), 4.87 – 4.80 (m, 1H), 3.72 (s, 3H), 3.68 – 3.38 (m, 4H), 3.33 – 3.21 (m, 2H), 3.18 – 3.11 (m, 1H), 3.11 – 2.89 (m, 7H), 2.53 – 2.34 (m, 15H), 2.19 – 1.62 (m, 6H), 1.56 – 1.41 (m, 1H), 1.15 – 0.98 (m, 1H), 0.86 – 0.72 (m, 1H); ^{13}C NMR (151 MHz, CD_2Cl_2, mixture of rotamers) δ 172.11, 170.28, 170.26, 156.00, 155.88, 155.60, 155.08, 154.92, 154.79, 154.73, 151.16, 151.12, 151.06, 151.04, 144.95, 141.26, 133.47, 133.35, 133.23, 132.10, 131.98, 131.62, 130.40, 130.33, 122.41, 122.36, 122.31, 122.23, 122.15, 74.98, 74.91, 74.76, 74.66, 53.63, 52.72, 47.85, 47.70, 47.45, 47.35, 47.20, 47.15, 46.71, 46.58, 41.17, 41.05, 37.47, 37.44, 37.30, 36.41, 36.36, 36.23, 36.15, 36.07, 35.64, 35.56, 35.52, 35.46, 35.30, 35.24, 34.98, 34.57, 32.31, 30.18, 30.13, 30.09, 29.91, 29.76, 29.72, 29.65, 29.61, 29.56, 29.45, 29.38, 27.57, 27.55, 25.89, 24.74, 24.55, 24.16, 23.09, 16.66, 14.59; HRMS [M+Na]$^+$ calcd. 786.3820 for $C_{40}H_{52}BF_2N_5O_7Na^+$, found 786.3849.

rTCO-DMEDA-Tyr-BODIPY (6)

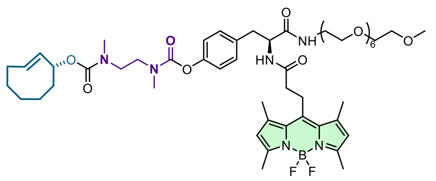

To a solution of **4** (21.7 mg, 28,4 µmol) in isopropanol (IPA) (2.84 mL) was added 1 M aqueous LiOH solution (2.84 mL) and the mixture was stirred for 20 min at rt, after which LCMS indicated complete saponification. A mixture of IPA and H_2O (4 mL, IPA/H_2O = 1:1) was added followed by addition of Amberlite IR120 (1.70 mL) and the suspension was rigorously stirred for 2 min. The solvent was removed upon filtration to obtain the free acid **5**, which was used without further purification. To a solution of crude **5** in DMF (1.6 mL) was added HBTU (21.5 mg, 56.8 µmol), DIPEA (24.6 µL, 18.4 mg, 142.0 µmol) and mPEG$_7$-NH$_2$ (18.4 µL, 19.3 mg, 56.8 µmol) and the mixture was stirred for 1 h at rt. LCMS indicated complete conversion to the desired product. Purification by preparative RP-HPLC (H_2O/MeCN gradient elution, 0.1% formic acid) afforded **6** as a red-brown solid (23.1 mg, 76%). ^{1}H NMR (600 MHz, CD_2Cl_2, mixture of rotamers) δ 7.22 – 7.10 (m, 2H), 7.06 – 6.93 (m, 2H), 6.57 (s, 2H), 6.08 (s, 2H), 5.87 – 5.71 (m, 1H), 5.59 – 5.46 (m, 1H), 4.64 (q, *J* = 7.1 Hz, 1H), 3.72 – 2.89 (m, 45H), 2.58 – 2.28 (m, 15H), 2.05 – 1.60 (m, 6H), 1.54 – 1.35 (m, 1H), 1.16 – 0.99 (m, 1H), 0.87 – 0.74 (m, 1H); ^{13}C NMR (151 MHz, CD_2Cl_2, mixture of rotamers) δ 170.80, 170.32, 170.30, 155.98, 155.86, 155.58, 155.12, 154.95, 154.82, 154.65, 150.96, 150.92, 150.87, 150.83, 145.17, 141.29, 134.24, 134.14, 134.03, 132.08, 132.00, 131.96, 131.62, 130.53, 130.46, 122.26, 122.20, 122.10, 74.97, 74.88, 74.75, 74.65, 72.28, 70.89, 70.87, 70.86, 70.84, 70.76, 70.62, 69.78, 59.00, 54.65, 54.57, 47.86, 47.70, 47.48, 47.34, 47.23, 47.16, 46.72, 46.61, 41.17, 41.06, 39.73, 38.40, 38.34, 38.29, 37.32, 36.41, 36.38, 36.23, 36.16, 35.65, 35.58, 35.55, 35.45, 35.32, 35.26, 34.98, 34.59, 29.45, 29.39, 24.75, 24.55, 24.25, 16.69, 14.58; HRMS [M+H]$^+$ calcd. 1071.5996 for $C_{54}H_{82}BF_2N_6O_{13}^+$, found 1071.6026.

Synthesis of tetrazines

DMT (**7**),[2] PA$_2$ (**8**)[3] and PymK (**9**)[4] were prepared according to known procedures.

Synthesis of sulfo-cTCO-DMEDA-caged CA4-prodrug 12

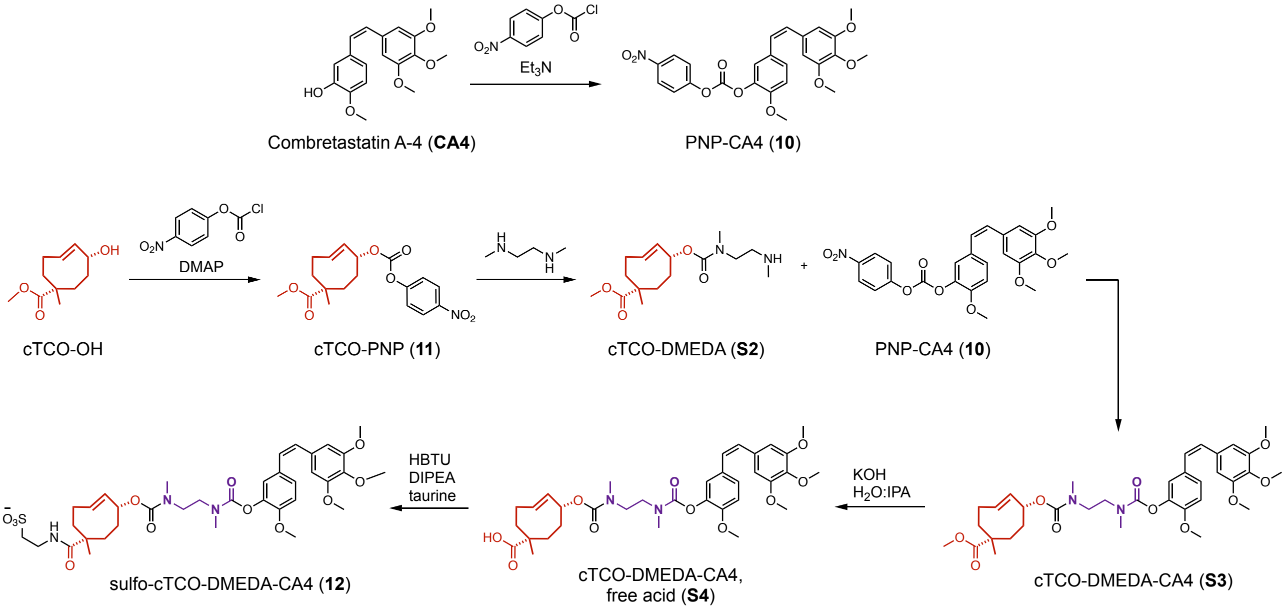

PNP-CA4 (10)

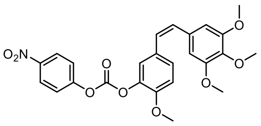

To a solution of **CA4** (27.0 mg, 85.4 µmol) in CH_2Cl_2 (1 mL) was added a solution of PNP chloroformate (20.7 mg, 102.5 µmol) in CH_2Cl_2 (1 mL) and Et_3N (28.6 µL, 20.7 mg, 205.0 µmol). The mixture was heated to reflux for 1 h, after which TLC indicated full consumption of the starting material. Purification by preparative HPLC (hexane/EtOAc gradient elution) afforded **10** as an off-white solid (38.0 mg, 92%). ^{1}H NMR (600 MHz, CD_2Cl_2) δ 8.32 – 8.27 (m, 2H), 7.48 – 7.42 (m, 2H), 7.21 (dd, *J* = 8.5, 2.1 Hz, 1H), 7.17 (d, *J* = 2.1 Hz, 1H), 6.95 (d, *J* = 8.5 Hz, 1H), 6.54 – 6.48 (m, 4H), 3.88 (s, 3H), 3.75 (s, 3H), 3.67 (s, 6H); ^{13}C NMR (151 MHz, CD_2Cl_2) δ 155.89, 153.57, 150.97, 150.37, 146.02, 139.75, 137.81, 132.64, 130.71, 130.47, 128.95, 128.42, 125.72, 122.76, 122.19, 112.77, 106.31, 60.84, 56.49, 56.19; HRMS [M+H]$^+$ calcd. 482.1446 for $C_{25}H_{24}NO_9^+$, found 482.1447.

cTCO-PNP (11)

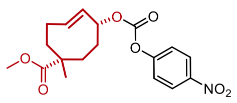

To an ice-cooled solution of cTCO-OH[5] (axial isomer, 80.0 mg, 404 µmol) in CH_2Cl_2 (2 mL), 4-(*N*,*N*-dimethylamino)pyridine (DMAP) (197.3 mg, 1615 µmol) in CH_2Cl_2 (1 mL) and PNP chloroformate (162.8 mg, 808 µmol) in CH_2Cl_2 (1 mL) were added and the mixture was stirred at rt for 4 h. Purification by column chromatography (CH_2Cl_2, isocratic elution) afforded **11** as an off-white solid (73.2 mg, 50%). ^{1}H NMR (600 MHz, CD_2Cl_2) δ 8.32 – 8.23 (m, 2H), 7.46 – 7.36 (m, 2H), 6.09 – 5.98 (m, 1H), 5.65 (dd, *J* = 16.8, 2.6 Hz, 1H), 5.26 (s, 1H), 3.62 (s, 3H), 2.37 – 2.25 (m, 2H), 2.17 (ddd, *J* = 14.0, 12.1, 5.0 Hz, 2H), 1.98 – 1.85 (m, 3H), 1.70 (ddd, *J* = 12.5, 9.0, 5.4 Hz, 1H), 1.13 (s, 3H); ^{13}C NMR (151 MHz, CD_2Cl_2) δ 180.00, 156.05, 151.98, 145.79, 133.33, 129.81, 125.62, 122.29, 77.48, 52.24, 45.15, 44.92, 35.95, 31.24, 30.90, 18.47; HRMS [M+H]$^+$ calcd. 364.1319 for $C_{18}H_{22}NO_7^+$, found 364.1319.

cTCO-DMEDA-CA4 (S3)

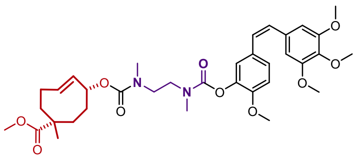

A solution of cTCO-PNP (**11**) (axial isomer, 24.2 mg, 66.6 µmol) in CH_2Cl_2 (3.8 mL) was added via syringe pump (30 µL/min) to a solution of DMEDA (71.7 µL, 58.7 mg, 666 µmol) in CH_2Cl_2 (1.15 mL) and DMF (0.37 mL) at 0 °C. Upon complete addition after 2 hours, stirring at 0 °C was continued for 1 h. Excess of DMEDA was removed under high vacuum (3 h) to obtain crude cTCO-DMEDA (**S2**), which was redissolved in CH_2Cl_2 (840 µL) and added to a solution of **10** (22.7 mg, 47.2 µmol) in DMF (420 µL) at rt. LCMS indicated complete conversion after a reaction time of 1 h. Purification by preparative HPLC (CH_2Cl_2/MeOH

gradient elution) afforded **S3** as a colorless oil (19.0 mg, 61%). ^{1}H NMR (600 MHz, CD_2Cl_2, mixture of rotamers) δ 7.12 (d, *J* = 8.5 Hz, 1H), 7.07 – 7.01 (m, 1H), 6.85 (d, *J* = 8.5 Hz, 1H), 6.52 (s, 2H), 6.50 – 6.42 (m, 2H), 5.94 – 5.76 (m, 1H), 5.67 – 5.54 (m, 1H), 5.15 (d, *J* = 17.7 Hz, 1H), 3.79 (d, *J* = 5.5 Hz, 3H), 3.75 (s, 3H), 3.68 (s, 6H), 3.64 – 3.42 (m, 7H), 3.12 – 2.89 (m, 6H), 2.30 – 1.58 (m, 8H), 1.14 – 1.05 (m, 3H); ^{13}C NMR (151 MHz, CD_2Cl_2, mixture of rotamers) δ 180.32, 180.24, 180.21, 155.75, 155.68, 155.40, 154.65, 154.60, 154.42, 153.47, 151.38, 151.33, 140.72, 140.65, 140.59, 137.68, 132.90, 132.87, 132.06, 131.91, 131.86, 131.81, 131.77, 130.32, 130.28, 129.72, 129.67, 128.96, 127.48, 127.43, 127.39, 124.26, 124.17, 124.10, 124.00, 112.41, 112.30, 112.20, 106.37, 73.22, 73.07, 73.01, 60.82, 56.33, 56.29, 56.24, 56.19, 52.15, 48.18, 47.96, 47.69, 47.60, 47.53, 47.15, 46.88, 46.68, 45.27, 45.22, 44.98, 36.21, 36.17, 35.79, 35.57, 35.45, 35.21, 34.95, 31.31, 31.22, 31.17, 18.37; HRMS $[M+H]^+$ calcd. 655.3225 for $C_{35}H_{47}N_2O_{10}^+$, found 655.3216.

sulfo-cTCO-DMEDA-CA4 (12)

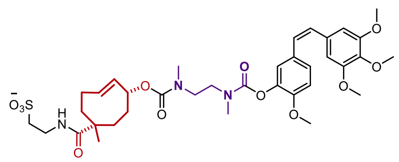

To a solution of **S3** (11.7 mg, 17.8 µmol) in IPA (0.5 mL) was added 5 M aqueous KOH solution (0.5 mL) and the mixture was stirred for 43 h at rt, after which LCMS indicated complete saponification. IPA/H_2O (2 mL, 1:1) and Amberlite IR120 (1.50 mL) were added, and the solution was rigorously stirred for 1 min. The mixture was filtered and concentrated to obtain free acid **S4**, which was used without further purification. To a solution of **S4** in DMSO (1.0 mL), HBTU (10.1 mg, 26.7 µmol), DIPEA (15.3 µL, 11.5 mg, 89.6 µmol) and taurine (6.2 mg, 49.8 µmol) were added, and the mixture was stirred at rt for 1 h. Purification by preparative RP-HPLC (phosphate buffer/MeCN gradient elution) and subsequent solid phase extraction afforded the sodium salt of **12** as a colorless oil (7.6 mg, 56%). Solid phase extraction was performed using a Strata® C18-E (55 µm, 70 Å), 50 mg / 1 mL tube (Phenomenex). The tube was conditioned with MeCN (600 µL) and equilibrated with H_2O (1800 µL). After preparative HPLC and evaporation of the solvent, the residue (containing the product and buffer salts) was dissolved in H_2O (300 µL) and loaded onto the tube. After washing with H_2O (600 µL), **12** was eluted with MeCN (600 µL) and the solvent was removed. ^{1}H NMR (600 MHz, DMSO-*d*$_6$, mixture of rotamers) δ 7.54 – 7.40 (m, 1H), 7.11 (dd, *J* = 8.5, 2.2 Hz, 1H), 7.07 – 6.92 (m, 2H), 6.62 – 6.53 (m, 2H), 6.53 – 6.42 (m, 2H), 5.80 – 5.65 (m, 1H), 5.65 – 5.52 (m, 1H), 5.00 (dd, *J* = 27.4, 15.3 Hz, 1H), 3.79 – 3.72 (m, 3H), 3.69 – 3.60 (m, 9H), 3.58 – 3.35 (m, 4H), 3.27 (s, 2H), 3.12 – 2.78 (m, 6H), 2.55 – 2.51 (m, 2H), 2.18 – 1.32 (m, 8H), 0.95 (d, *J* = 23.9 Hz, 3H); ^{13}C NMR (151 MHz, DMSO-*d*$_6$, mixture of rotamers) δ 178.99, 178.90, 154.69, 154.64, 154.46, 154.40, 153.57, 153.43, 153.32, 153.03, 152.59, 150.71, 150.63, 139.69, 139.65, 136.77, 136.71, 132.05, 132.01, 131.65, 131.33, 130.97, 130.90, 130.83, 130.79, 129.20, 129.14, 129.07, 128.35, 128.28, 126.81, 126.68, 123.47, 123.21, 112.48, 112.32, 105.93, 103.66, 72.38, 72.20, 72.15, 60.05, 60.01, 55.85, 55.80, 55.62, 55.59, 50.08, 46.79, 46.69, 46.45, 46.34, 46.27, 45.99, 45.65, 45.58, 45.01, 43.48, 43.44, 35.68, 35.35, 35.30, 35.25, 35.20, 34.82, 34.70, 34.65, 34.60, 34.40, 34.20, 33.97, 30.85, 30.75, 30.63, 30.58, 17.82; HRMS $[M+H]^+$ calcd. 748.3110 for $C_{36}H_{50}N_3O_{12}S^+$, found 748.3095.

3 Release Experiments

Instrument and solvents

Reaction monitoring of release experiments was performed on a Nexera X2® UHPLC system (Shimadzu®) with a temperature-controlled autosampler at 37 °C. For acidic HPLC conditions, the aqueous solvent was prepared by addition of 2.5 mL of neat formic acid to 2.5 L of HPLC-water to yield a final concentration of 0.1% formic acid. For buffered HPLC conditions, the aqueous solvent was prepared by addition of 625 µL of 10 M ammonium formate (BioUltra, Sigma-Aldrich) to 2.5 L of HPLC-grade water followed by adjusting the pH to 8.4 by addition of 25% aqueous ammonia (for HPLC, LiChropur, Merck). Since its pH declines over time, this volatile buffer was freshly prepared each day. HPLC-grade acetonitrile was used without any additives.

Stock solutions

Stock solutions of rTCO-DMEDA-Tyr-BODIPY (**6**) and sulfo-cTCO-DMEDA-CA4 (**12**) were prepared at a concentration of 20 mM in DMSO. Tetrazine stock solutions of DMT (**7**), PA_2 (**8**) and PymK (**9**) were prepared at a concentration of 10 mM in DMSO.

Release kinetics measurements

rTCO-DMEDA-Tyr-BODIPY (**6**): The stock solution of **6** was diluted with PBS to a concentration of 100 µM in an HPLC vial (2.5 µL 20 mM TCO stock, 497.5 µL PBS). Tetrazine stock solutions were diluted with PBS to a concentration of 200 µM in an HPLC vial (2.5 µL Tz stock, 122.5 µL PBS), and the click-to-release reaction was initiated by addition of the diluted TCO solution (125 µL) to obtain starting concentrations of 50 µM TCO and 100 µM Tz. The samples were immediately incubated at 37 °C in the autosampler and subjected to serial HPLC analysis. All measurements were conducted in triplicates.

sulfo-cTCO-DMEDA-CA4 (**12**): The stock solution of **12** was diluted with DMSO (30 µL TCO stock, 30 µL DMSO) to give a 10 mM stock solution. Tz stock solution (10 µL) was added to PBS (985 µL, containing 8.6% DMSO), and the click-to-release reaction was initiated by addition of the stock solution of **12** (5.24 µL) to obtain starting concentrations of 50 µM TCO and 100 µM Tz (in 10% DMSO/PBS). The samples were immediately incubated at 37 °C in the autosampler and subjected to serial HPLC analysis in intervals of 30 min. All measurements were conducted in triplicates.

Determination of exact TCO stock concentrations

The exact TCO stock concentrations were determined by absorbance titration (535 nm) with a freshly prepared stock solution of 3,6-bis(2-pyridyl)tetrazine **$2Pyr_2$** (Sigma Aldrich) in DMSO using a Thermo Fisher Scientific NanoDrop One^C Microvolume UV-Vis Spectrophotometer in cuvette mode at 25 °C. The TCO stock solution (20 mM) was diluted with DMSO to reach a concentration of 1 mM, and then spiked with an excess of **$2Pyr_2$** stock solution (20.4 mM). Upon IEDDA reaction, the remaining tetrazine absorbance at 535 nm was measured. This procedure was repeated twice (standard addition) to determine the exact TCO stock concentration.

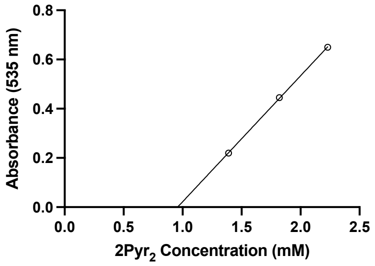

Analytical HPLC analysis

rTCO-DMEDA-Tyr-BODIPY (**6**): PDA data was collected for all samples and used to identify all signals showing a characteristic BODIPY absorption. Relative quantification of reactants, intermediates and products was done using extracted chromatograms (wavelength: 500 nm).

sulfo-cTCO-DMEDA-CA4 (**12**): PDA data was collected for all samples. Relative quantification of intermediates and products was done using extracted chromatograms (wavelength: 254 nm). In addition, released **CA4** was quantified via external calibration.

External CA4 calibration
A **CA4** stock solution (20 mM) was prepared in DMSO and diluted with PBS (containing 10% DMSO) to reach a concentration of 100 µm. **CA4** standard solutions (1 µM – 75 µM) were prepared by serial dilution into PBS (containing 10% DMSO). All measurements were conducted in triplicates.

Selected chromatograms and MS data

Tz-triggered cleavage of rTCO-DMEDA-Tyr-BODIPY (6)

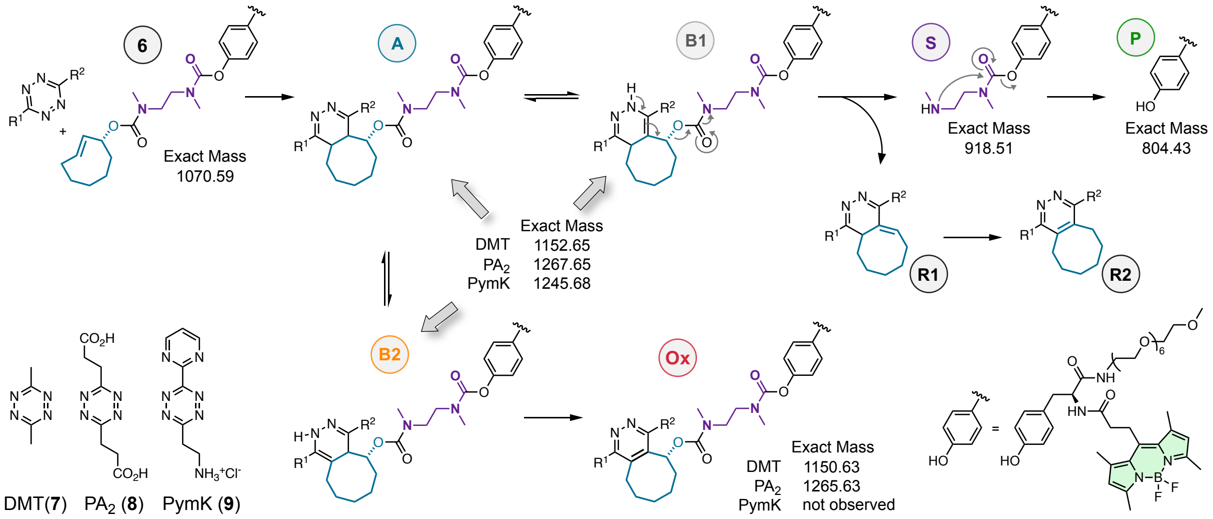

rTCO-DMEDA-Tyr-BODIPY (6) + DMT (7), 65 min reaction time, 15% phenol release

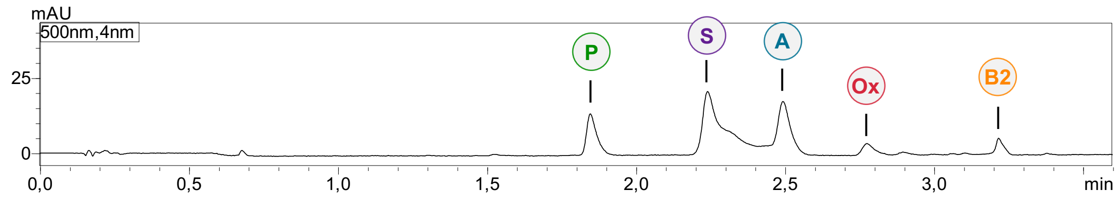

HPLC gradient (% acetonitrile in 2.5 mM ammonium formate buffer, pH 8.4) 0 min: 2%, 0.25 min: 2%, 0.27 min: 35%, 2.50 min: 50%, 3.56 min: 85%.

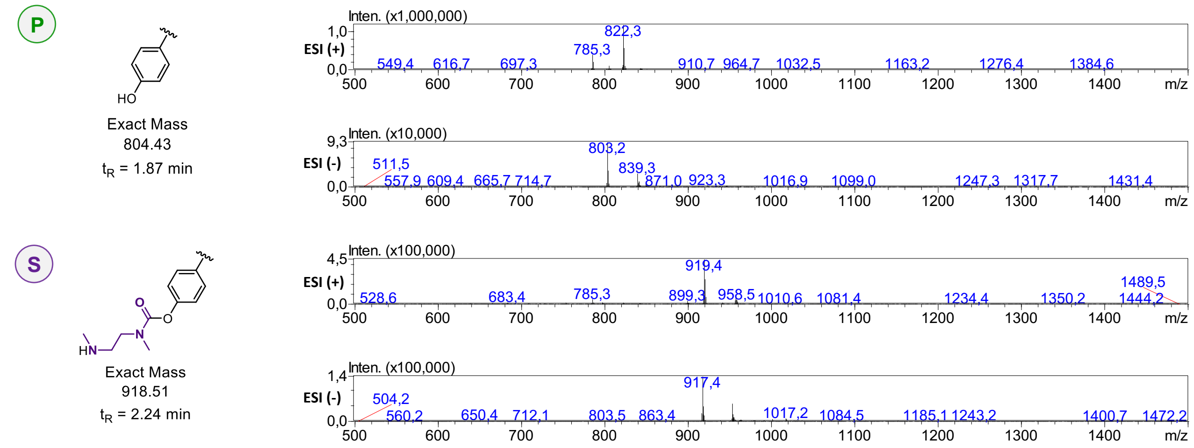

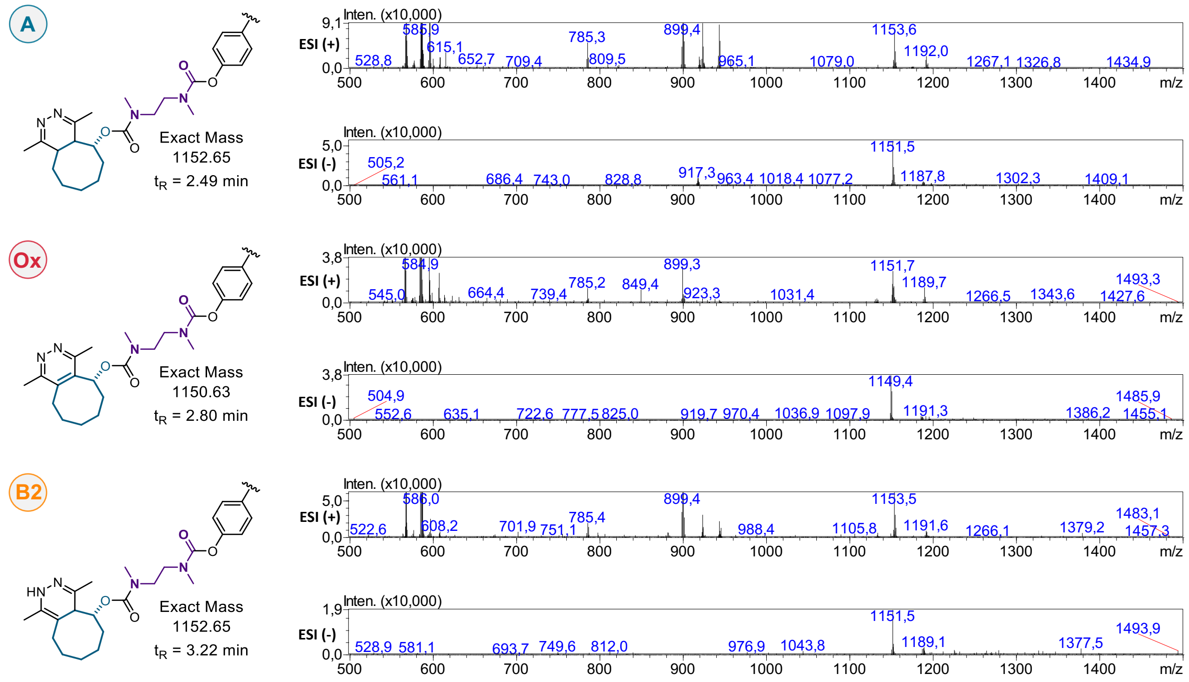

rTCO-DMEDA-Tyr-BODIPY (6) + PA$_2$ (8), 4h 5 min reaction time, 52% phenol release

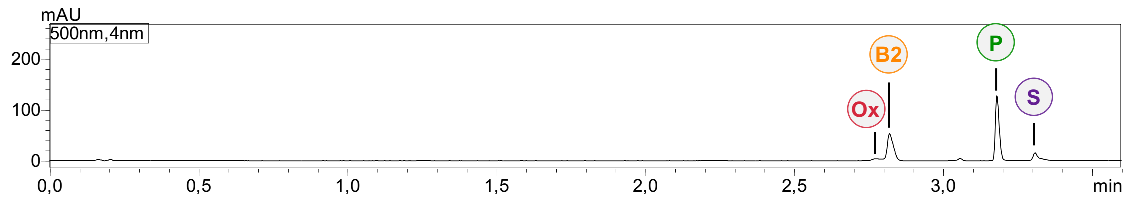

HPLC gradient (% acetonitrile in 2.5 mM ammonium formate buffer, pH 8.4) 0 min: 5%, 0.15 min: 5%, 0.17 min: 20%, 2.30 min: 25%, 3.51 min: 98%, 3.76 min: 98%.

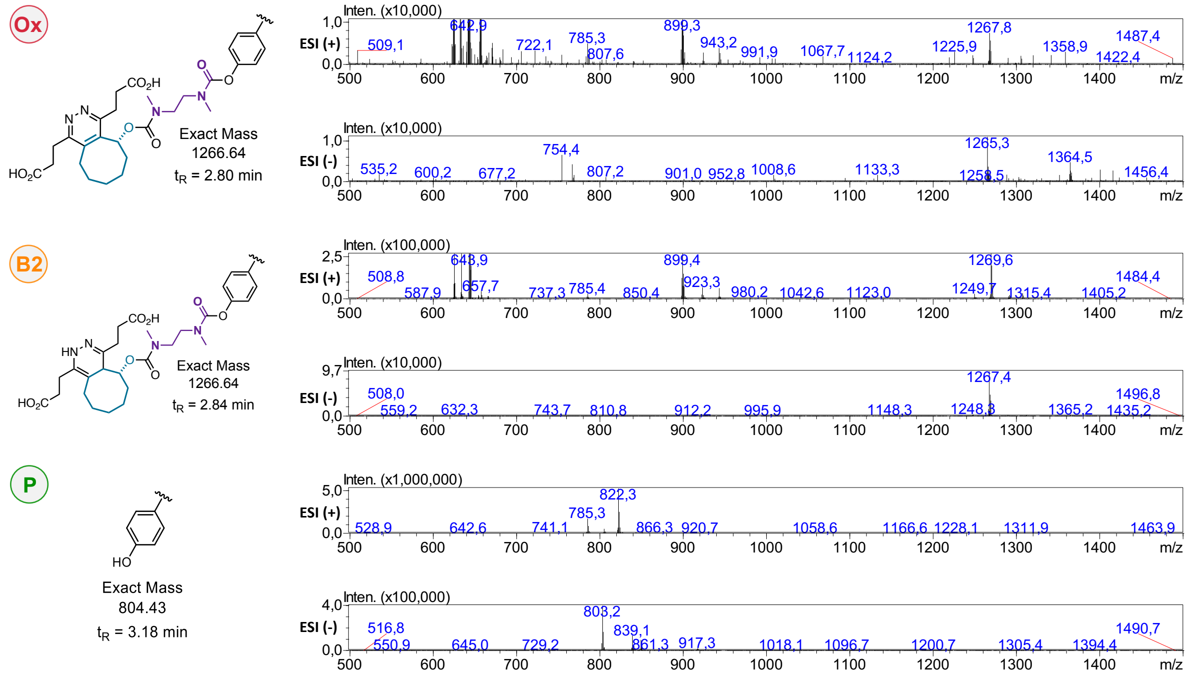

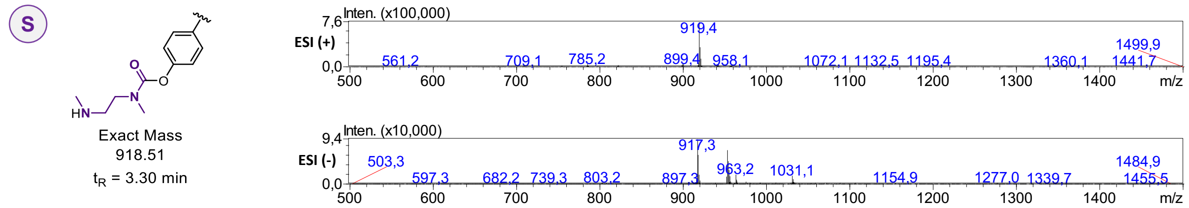

rTCO-DMEDA-Tyr-BODIPY (6) + PymK (9), 65 min reaction time, 44% phenol release

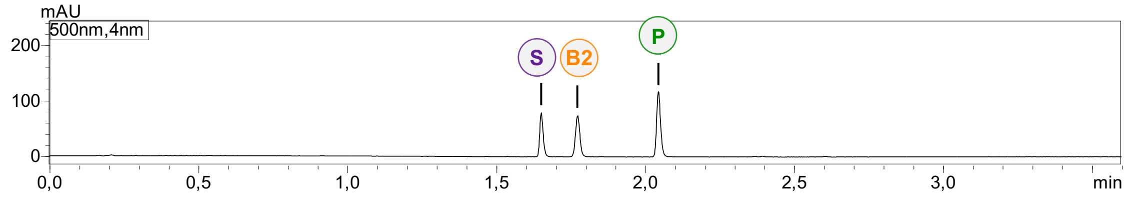

HPLC gradient (% acetonitrile in 0.1% formic acid) 0 min: 5%, 0.15 min: 5%, 3.20 min: 98%, 3.50 min: 98%.

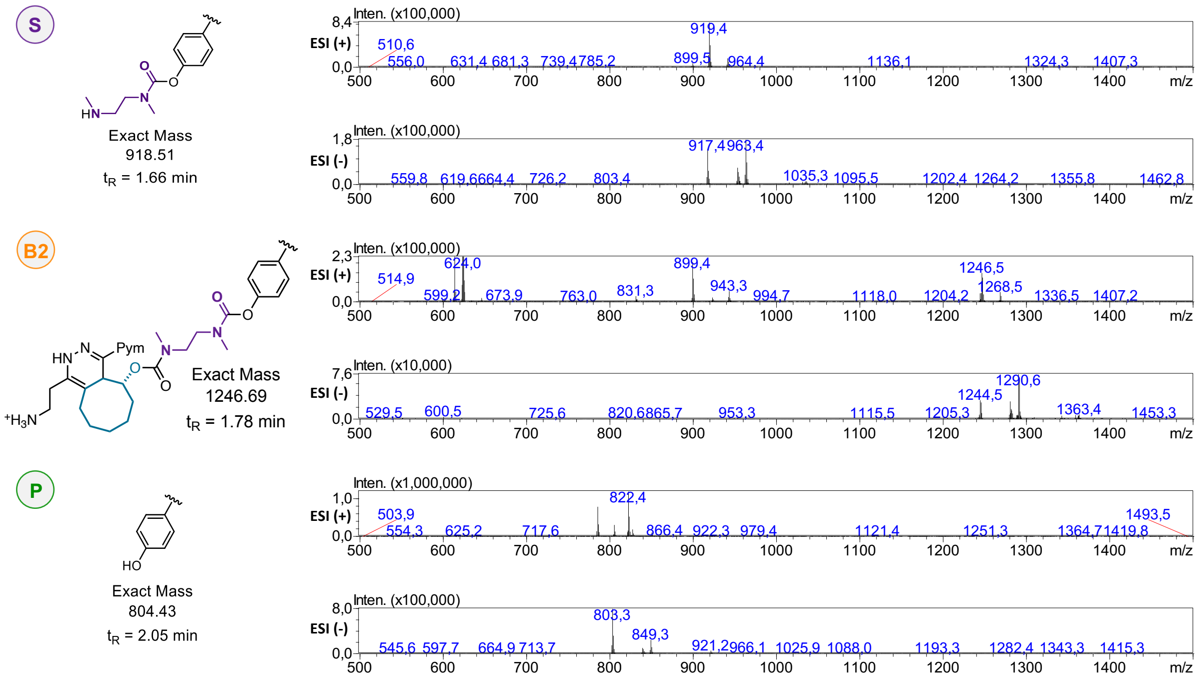

Tz-triggered cleavage of sulfo-cTCO-DMEDA-CA4 (12)

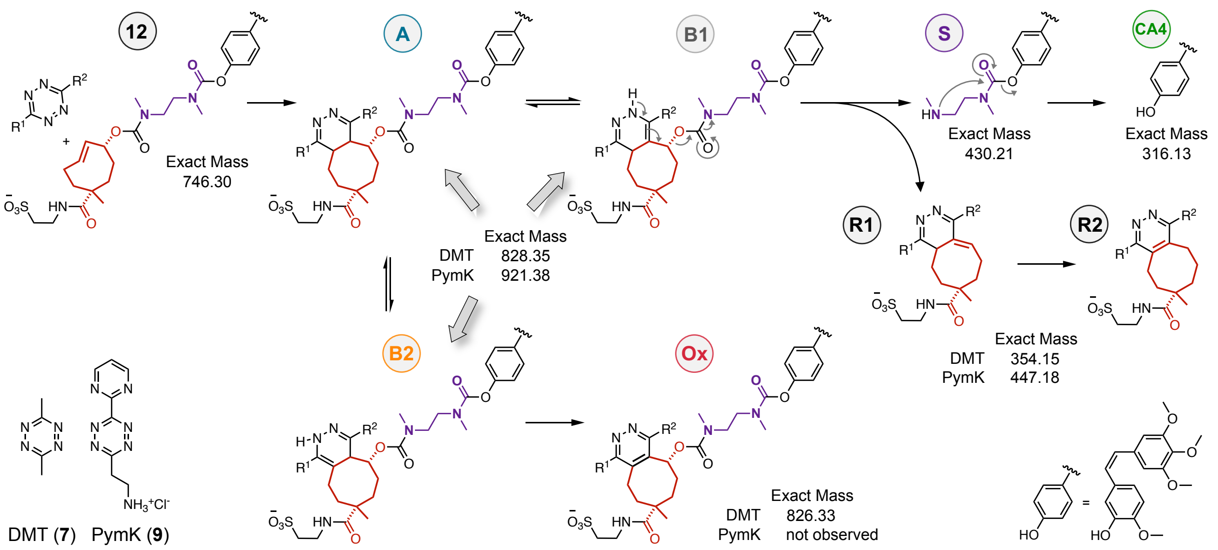

sulfo-cTCO-DMEDA-CA4 (12) + DMT (7), 35 min reaction time, 47% phenol release

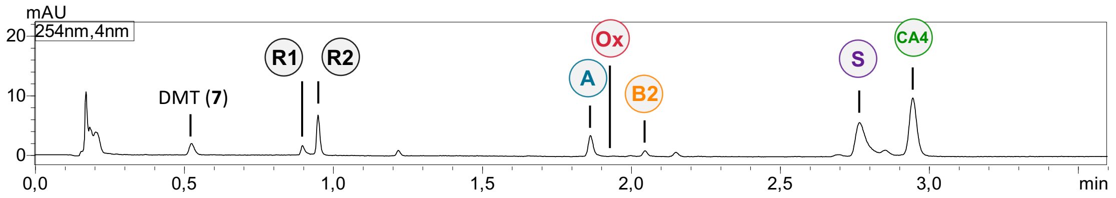

HPLC gradient (% acetonitrile in 2.5 mM ammonium formate buffer, pH 8.4) 0 min: 5%, 0.15 min: 5%, 1.20 min: 30%, 3.45 min: 50%, 3.56 min: 50%.

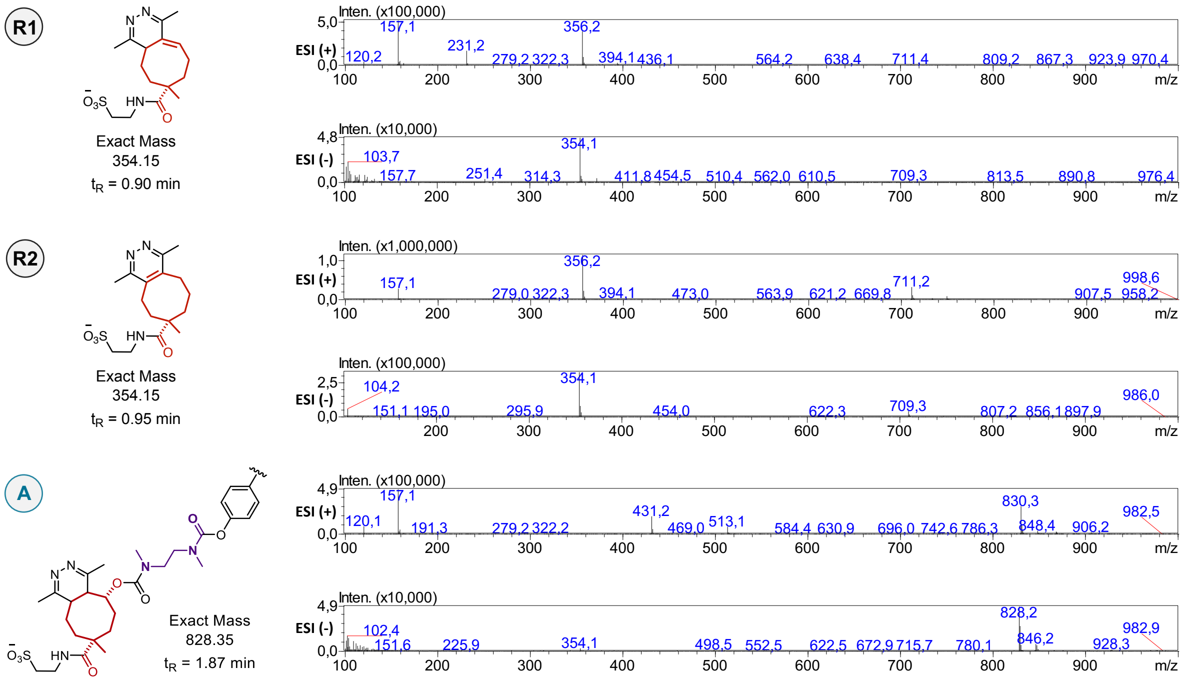

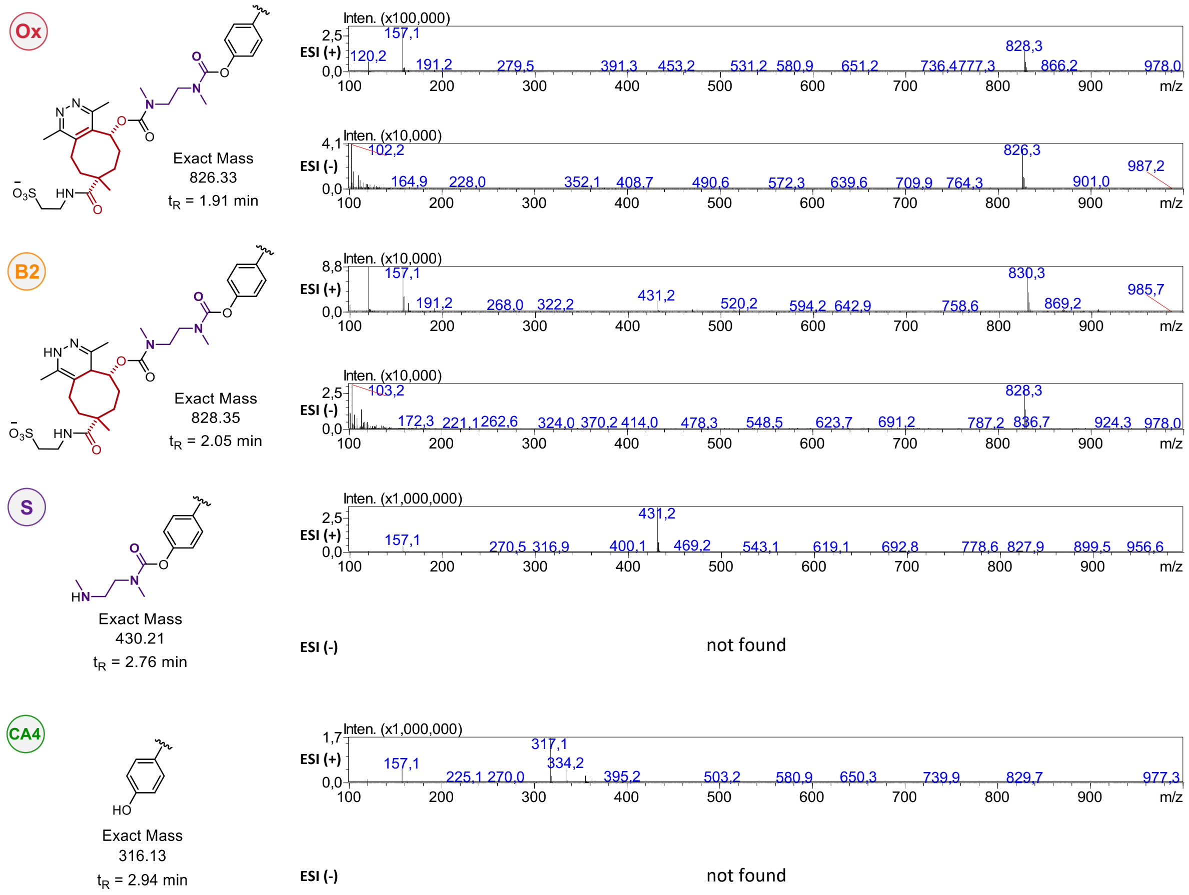

sulfo-cTCO-DMEDA-CA4 (12) + PymK (9), 35 min reaction time, 31% phenol release

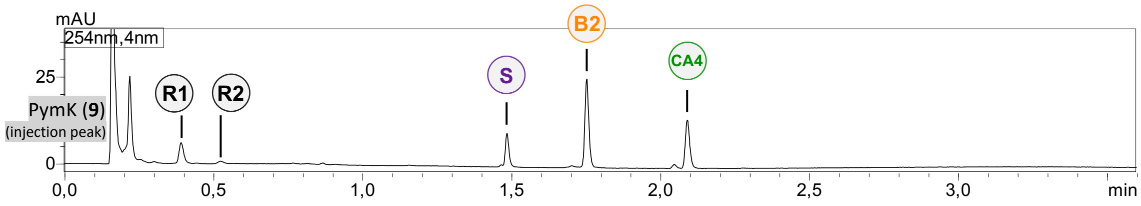

HPLC gradient (% acetonitrile in 0.1% formic acid) 0 min: 5%, 0.15 min: 5%, 3.20 min: 98%, 3.50 min: 98%.

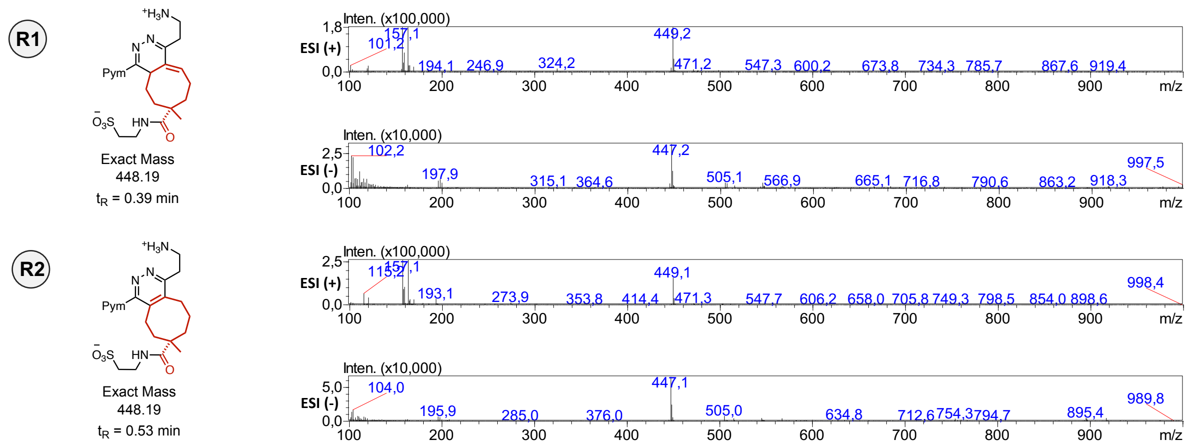

S

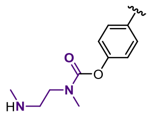

Exact Mass
430.21
t_R = 1.49 min

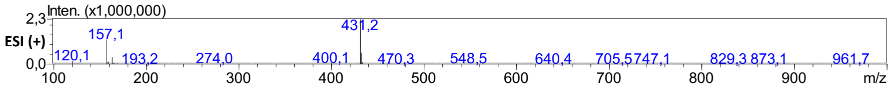

ESI (-) not found

B2

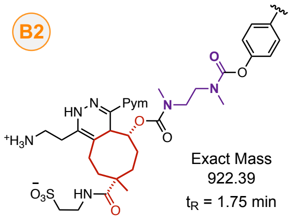

Exact Mass
922.39
t_R = 1.75 min

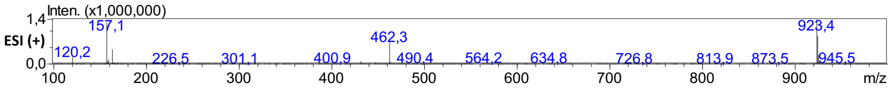

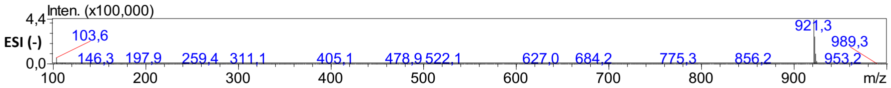

CA4

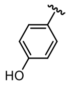

Exact Mass
316.13
t_R = 2.09 min

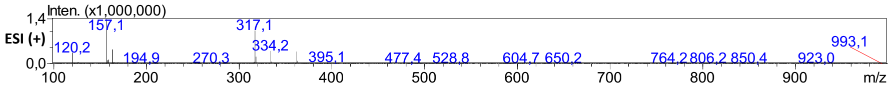

ESI (-) not found

Release performance of sulfo-cTCO-DMEDA-CA4 (12)

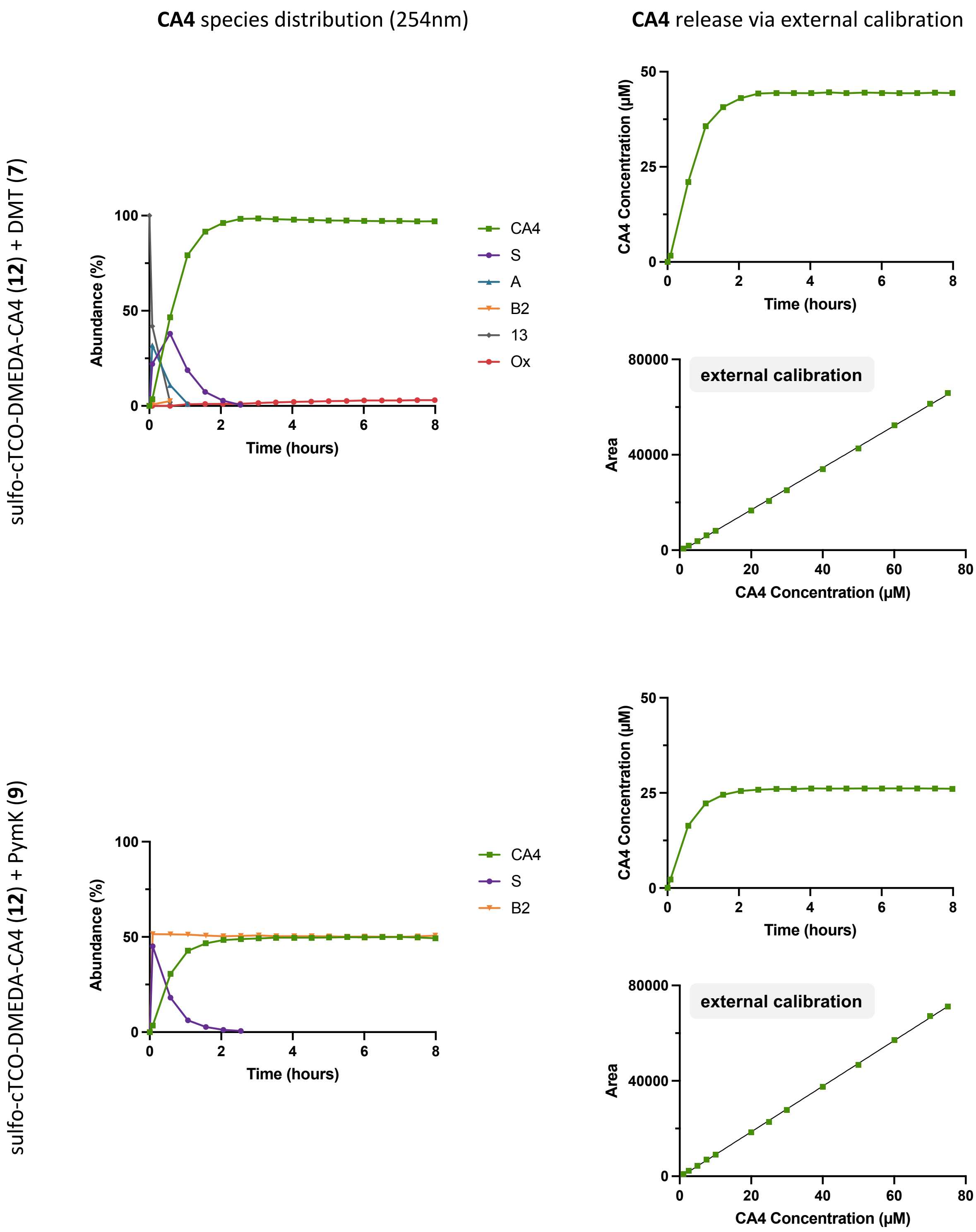

Release performance of sulfo-cTCO-DMEDA-CA4 (**12**) (50 µM) upon reaction with DMT (**7**) or PymK (**9**) (100 µM) in PBS at 37 °C: Relative quantification was done using extracted chromatograms (254 nm, left). In addition, released **CA4** was quantified via external calibration (right) to correct for the different absorption of intermediates/products at 254 nm.

4 Click Kinetics

A stock solution of **rTCO-PEG$_4$**[3] in DMSO was prepared at an approximate concentration of 20 mM. The exact concentration was determined by absorbance titration with DMT (**7**) (extinction coefficient 510 $M^{-1}cm^{-1}$ at 520 nm), quantifying the decrease in tetrazine absorbance upon reaction with TCO. The initial DMSO stock was diluted with PBS to prepare solutions for stopped-flow analysis at a final TCO concentration of 1 mM.

20 mM stock solutions of DMT (**7**), PA$_2$ (**8**), and PymK (**9**) in DMSO were prepared. Serial dilution into PBS gave solutions for stopped-flow analysis at a Tz concentration of 100 µM.

Stopped-flow measurements were performed using an SX20-LED stopped-flow spectrophotometer (Applied Photophysics) equipped with a 535nm LED (optical pathlength 10 mm, full width half-maximum 34 nm) to monitor the characteristic tetrazine visible light absorbance (520-540 nm). The reagent syringes were loaded with tetrazine and TCO solutions and the instrument was primed. Measurements were done in sextuplicate for each Tz. Reactions were conducted at 37 °C and recorded automatically at the time of acquisition.

Data sets were analyzed by fitting an exponential decay using Prism 6 (GraphPad) to calculate the observed pseudo-first-order rate constants that were converted into second-order rate constants (Table S1) by dividing through the TCO concentration.

Table S1. Second-order rate constants (k_2) of Tz with rTCO-PEG$_4$ (PBS, 37 °C), in comparison to the observed release yields.

Tz	k_2 ($M^{-1}s^{-1}$) with **rTCO-PEG$_4$**	Release yield with **6**	Release yield with **12**
DMT (**7**)	82 ± 9	89%	98%
PA$_2$ (**8**)	12 ± 1	92%	n.d.
PymK (**9**)	1420 ± 160	70%	50%

5 Cell Viability Assays

HT1080 human fibrosarcoma cells (ATCC) were cultivated in EMEM (Minimum Essential Medium Eagle, with Earle's salts, L-glutamine and sodium bicarbonate; Sigma Aldrich) supplemented with 10% fetal bovine serum and 1% antibiotic/antimycotic solution (100X, Sigma-Aldrich) at 37 °C and 5% CO_2. HT1080 cells were seeded into 96-well plates (triplicates for each group) at 10.000 cells per well and allowed to grow overnight.

The medium was removed and a dilution series of sulfo-cTCO-DMEDA-CA4 (**12**) or the parent drug **CA4** in growth medium (10 µM, 2 µM, 0.4 µM, 0.08 µM, 0.016 µM, 0.0032 µM, 0.00064 µM, 0.000128 µM, 0.0000256 µM) was added to the cells (0.1% final DMSO concentration). For release experiments the same concentrations of **12** were used, while a stock solution of DMT (**7**) or PymK (**9**) was added to obtain final concentration of 5 µM of the respective tetrazine. Incubation was carried out for 72 h.

Cell viability was assessed by replacing the medium with 100 µL of PrestoBlue solution (Invitrogen, 1:9 in growth medium) followed by incubation for 30 minutes at 37 °C. Read-out of the fluorescence signal was carried out using a PerkinElmer EnSpire Multimode Plate Reader and data processing was done in GraphPad Prism.

Following the same procedure, cells were treated with DMT (**7**), PymK (**9**), or 1,3-dimethylimidazolidin-2-one (= byproduct of the self-immolation process) with concentrations of up to 10 µM, revealing no significant effect on cell viability.

6 Cell Imaging

HT1080 cells were seeded into a 96-well plate at 3,000 cells per well and allowed to grow overnight. The medium was removed, and the cells were treated with a 200 nM solution of sulfo-cTCO-DMEDA-CA4 (**12**) in media. *In situ* click-to-release was initiated by addition of DMT (**7**) at a final concentration of 10 µM. As controls, cells were left untreated or incubated with either the parent drug **CA4** (200 nM) or 10 µM DMT (**7**). After an incubation time of 6 h cells were stained with SiR-tubulin (a fluorogenic, cell permeable and highly specific probe for microtubules).[6] An 11X stock solution of the probe was directly added to the growth medium to obtain a final concentration of 1 µM and incubation was carried out for 1 h. Subsequently, the medium was removed and cells were stained with Hoechst 33342 nuclear dye (Invitrogen, 5 µM in growth medium) for 10 minutes and washed once with PBS. Multichannel imaging of the cells was carried out in FluoroBrite DMEM medium (Gibco) on an Olympus IX82 microscope (Fig. S1).

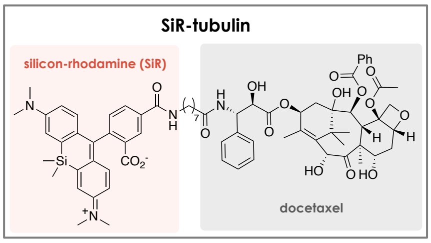

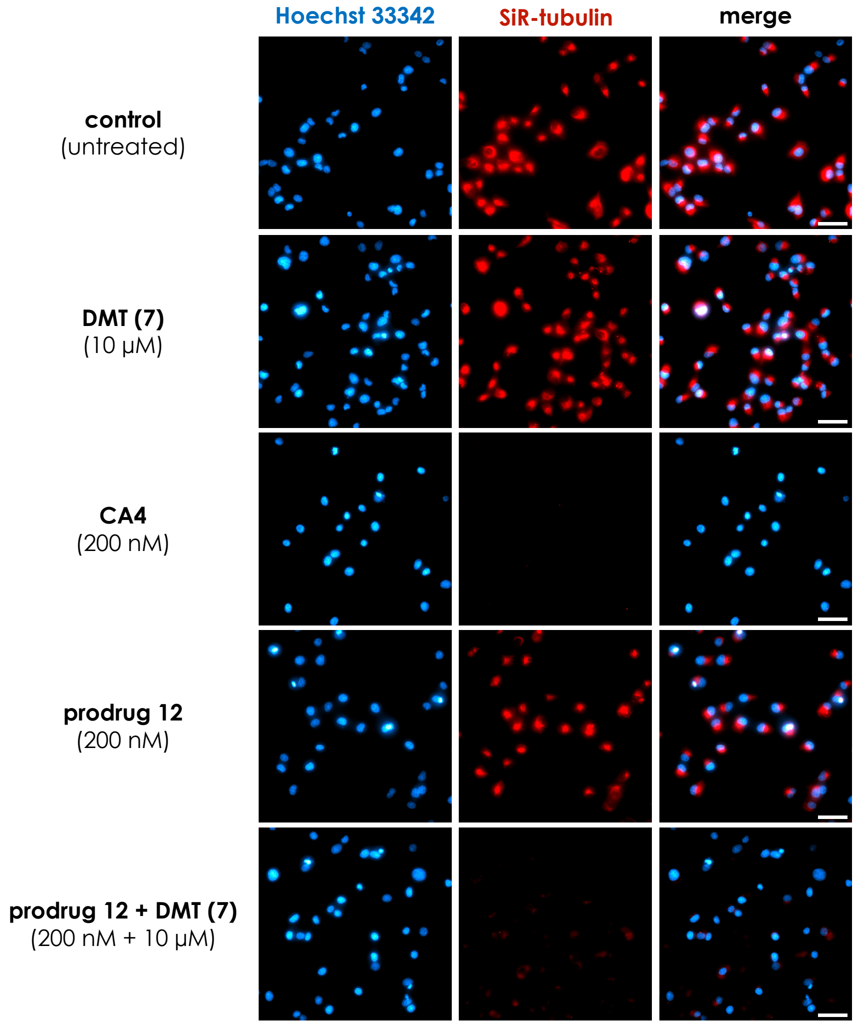

Figure S1. Cell imaging via fluorescence microscopy (scale bars: 50 µm) upon staining with Hoechst 33342 (blue, nuclei) and SiR-tubulin[6] (red, microtubules) shows comparable depletion of tubulin signals after 6 h treatment with **CA4** (200 nM) or bioorthogonal activation of prodrug **12** (200 nM) by *in situ* reaction with DMT (**7**). No significant change (compared to untreated cells) was observed after treatment with DMT (**7**) or prodrug **12**.

7 Prodrug Stability

Prodrug **12** was incubated in (i) PBS and (ii) cell growth medium (DMEM + 10% fetal bovine serum) at 37 °C at a concentration of 100 µM (1 µL 10 mM stock in DMSO + 99 µL PBS/medium). Samples in PBS were analyzed by serial HPLC measurements (n=3) for 120 h. No degradation of the DMEDA bis(carbamate)-linkage and <5% isomerization of the cTCO linker was observed.

Aliquots (50 µL) of samples in cell growth medium were diluted with ice cold MeCN (200 µL) followed by centrifugation at 14,000 rpm for 8 min at 4 °C. A sample of **CA4** (100 µM in cell growth medium) was prepared following the same procedure as a control. HPLC analysis of the supernatants (n=3) revealed 33.0 ± 0.2% *trans*-to-*cis* isomerization of the cTCO linker after 72 h and 43.6 ± 1.0% after 120 h (as verified by addition of **2Pyr$_2$**), but **no release of CA4**, confirming integrity of the DMEDA bis(carbamate)-linkage (Fig. S2).

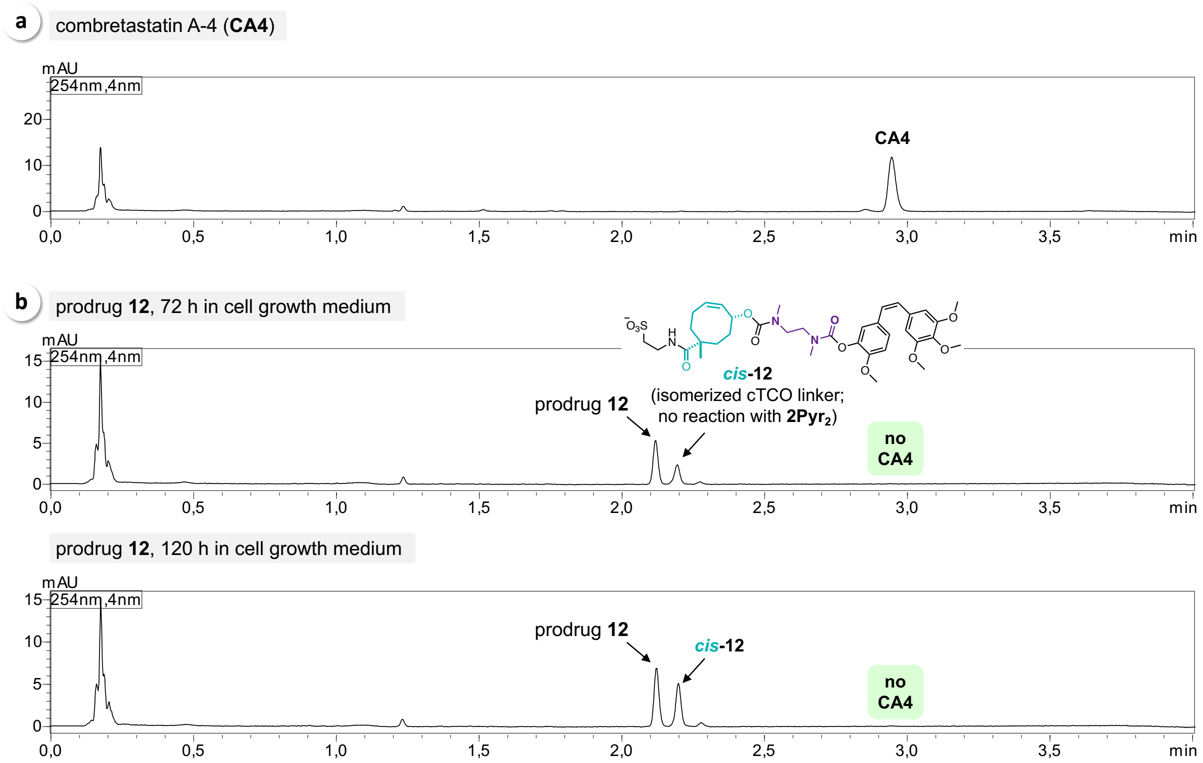

Figure S2. HPLC chromatograms (254 nm) of (a) **CA4** (control), and (b) prodrug **12** incubated in cell growth medium (DMEM + 10% fetal bovine serum) at 37 °C for 72 h and 120 h. HPLC gradient (%MeCN in 2.5 mM ammonium formate buffer, pH 8.4): 0 min 5% - 0.15 min, 5% - 1.20 min, 30% - 3.45 min, 50% - 3.56 min, 50%.

8 NMR Spectra, Chromatograms and MS Data

S1, ^{1}H NMR

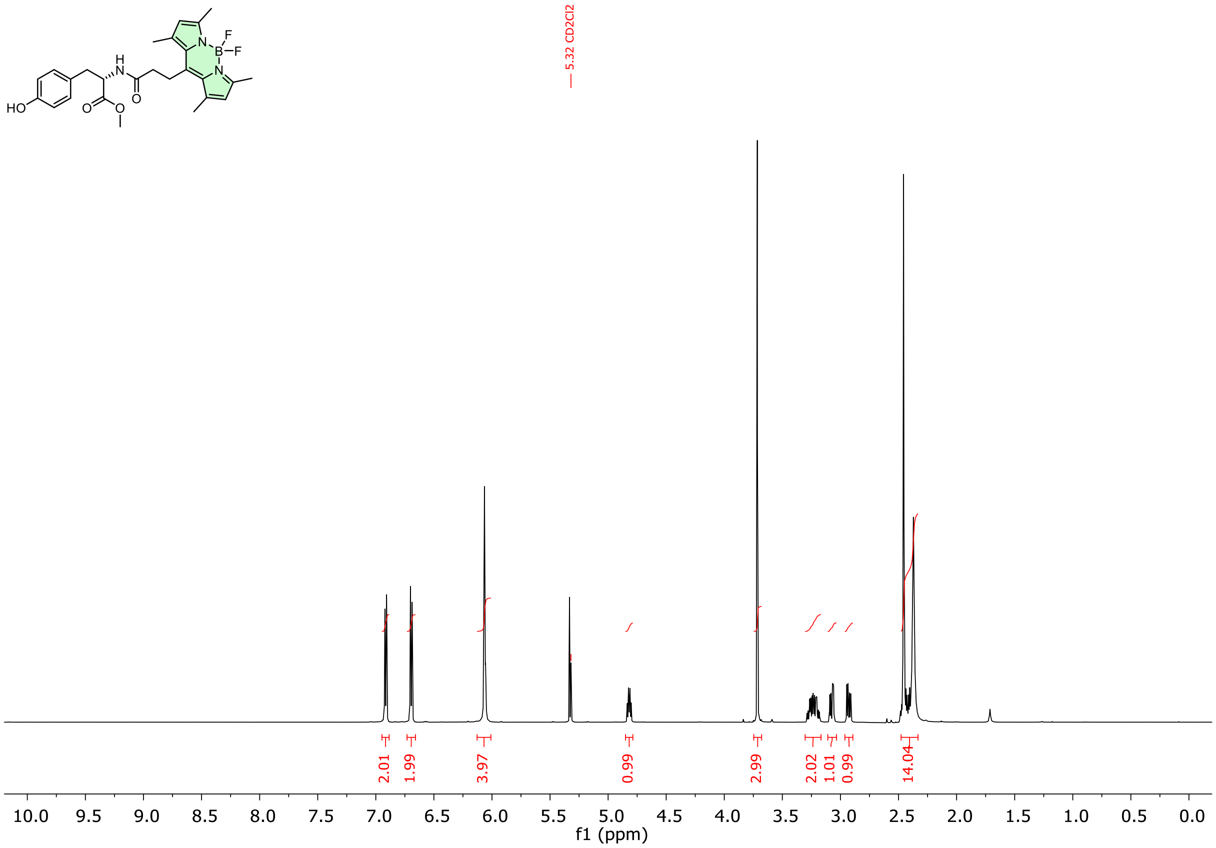

S1, ^{13}C NMR

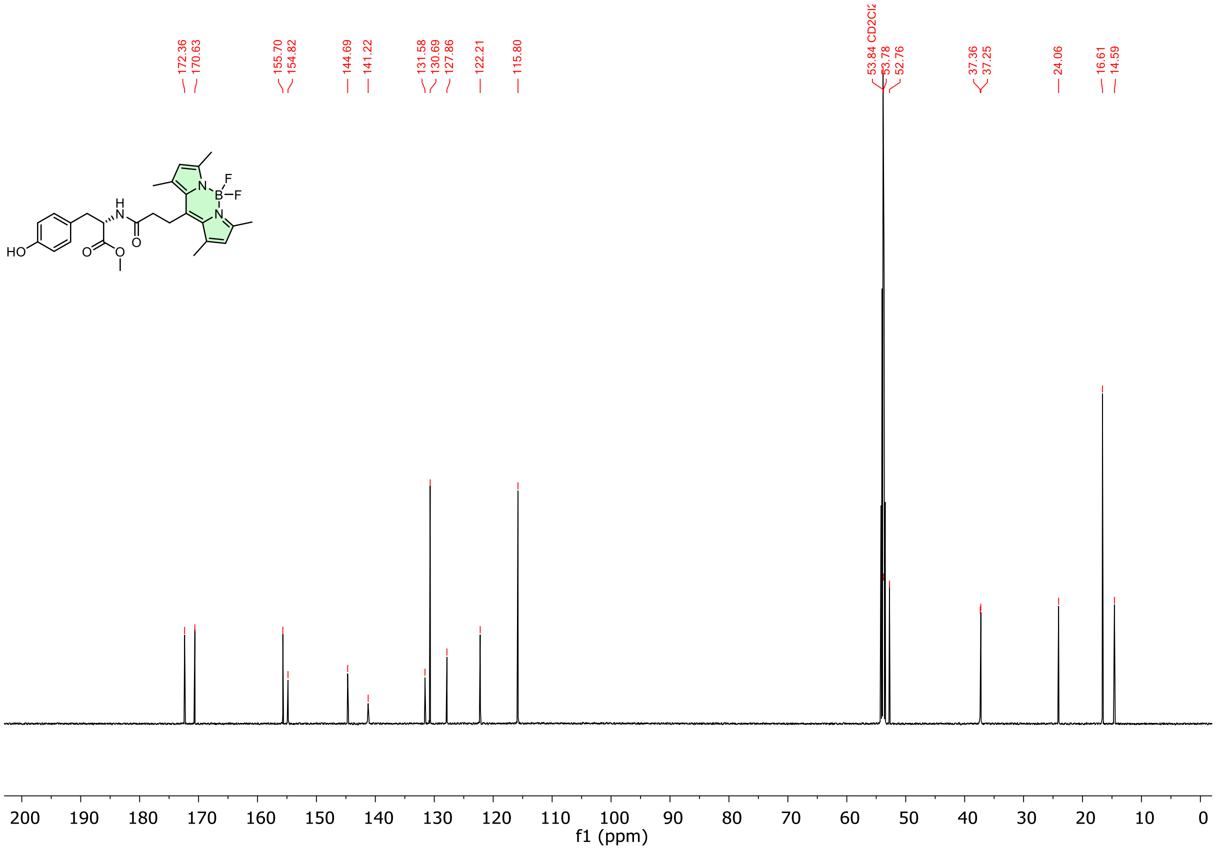

S1, HPLC (acidic conditions)

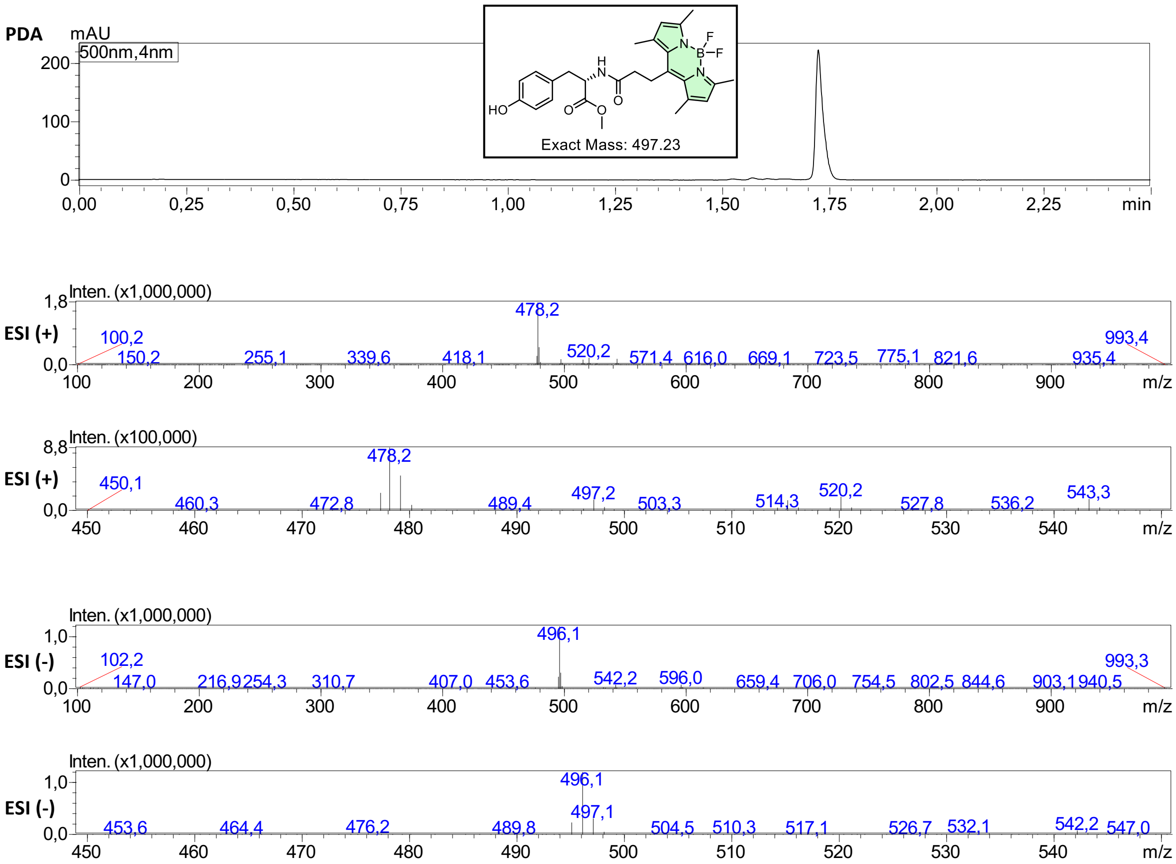

3, ^{1}H NMR

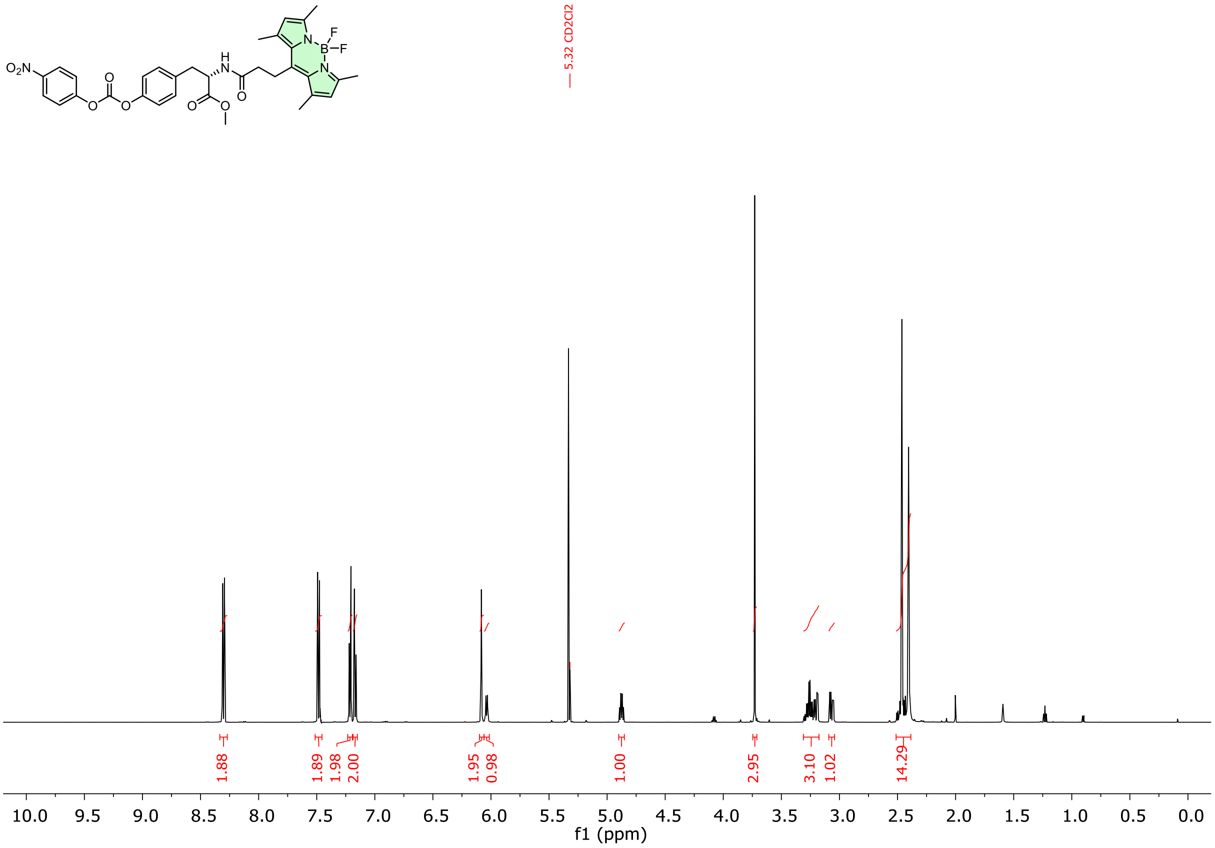

3, ^{13}C NMR

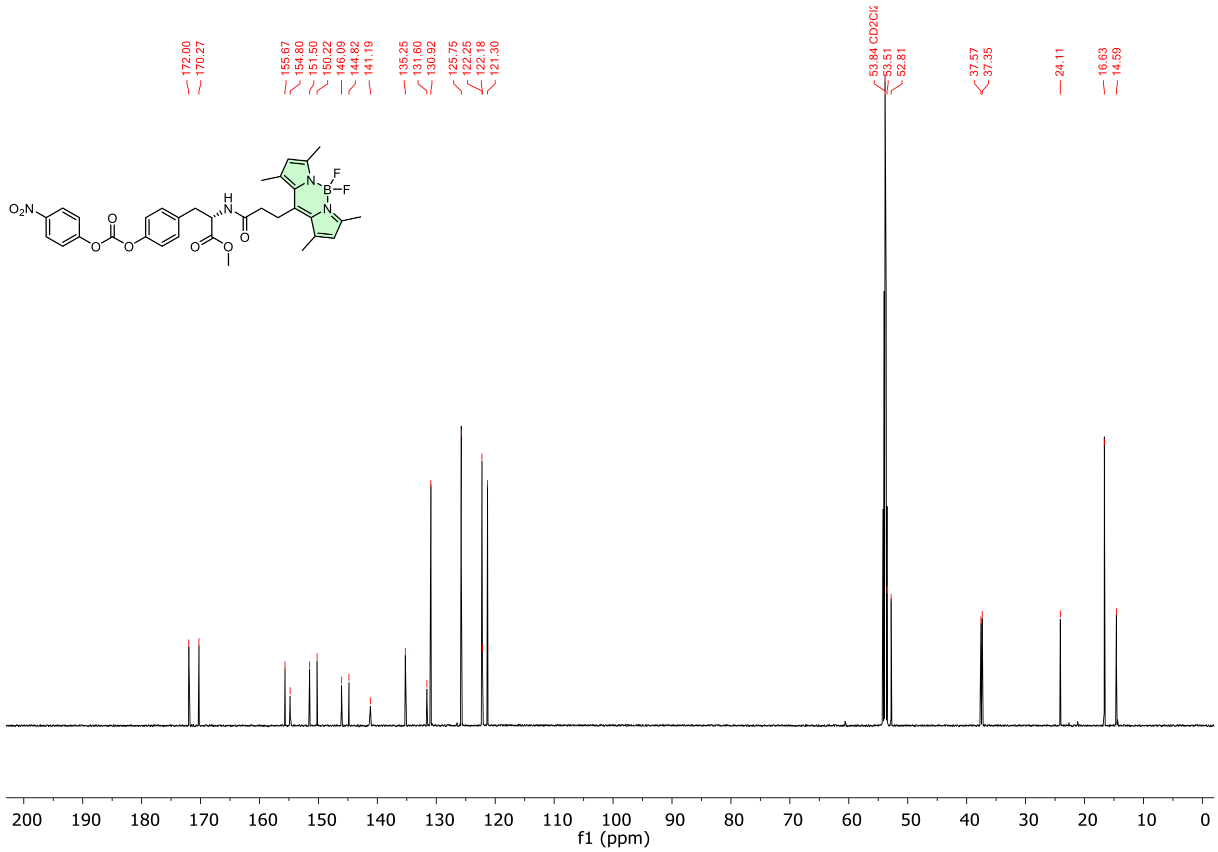

3, HPLC (acidic conditions)

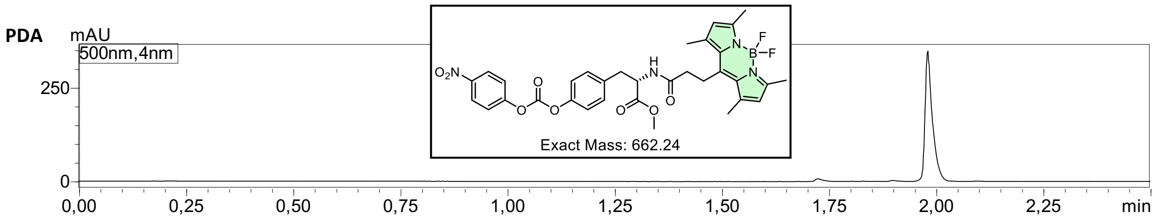

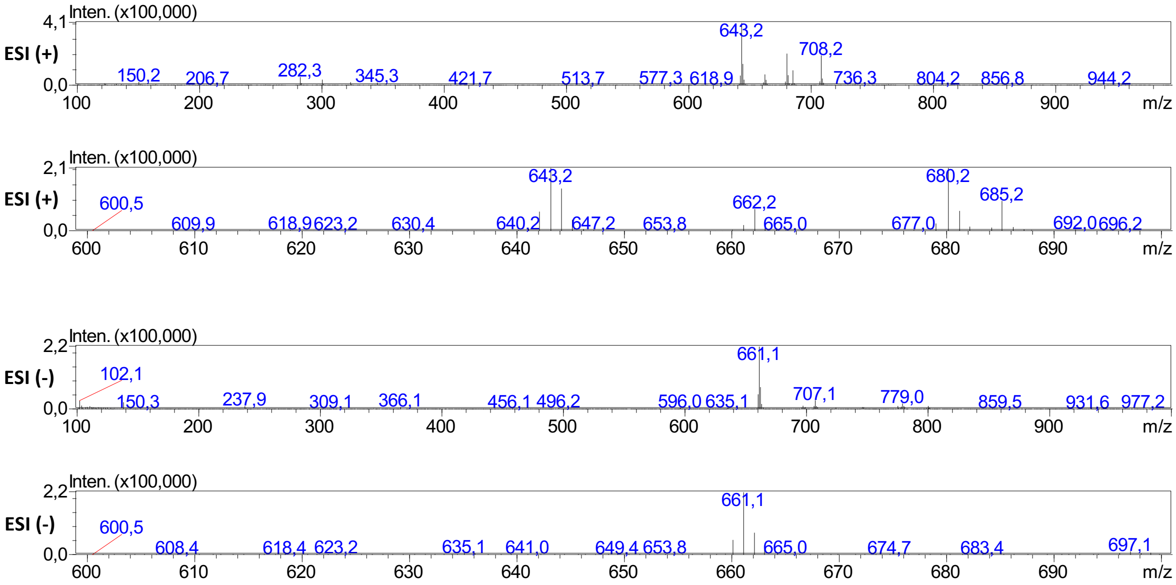

4, ^{1}H NMR

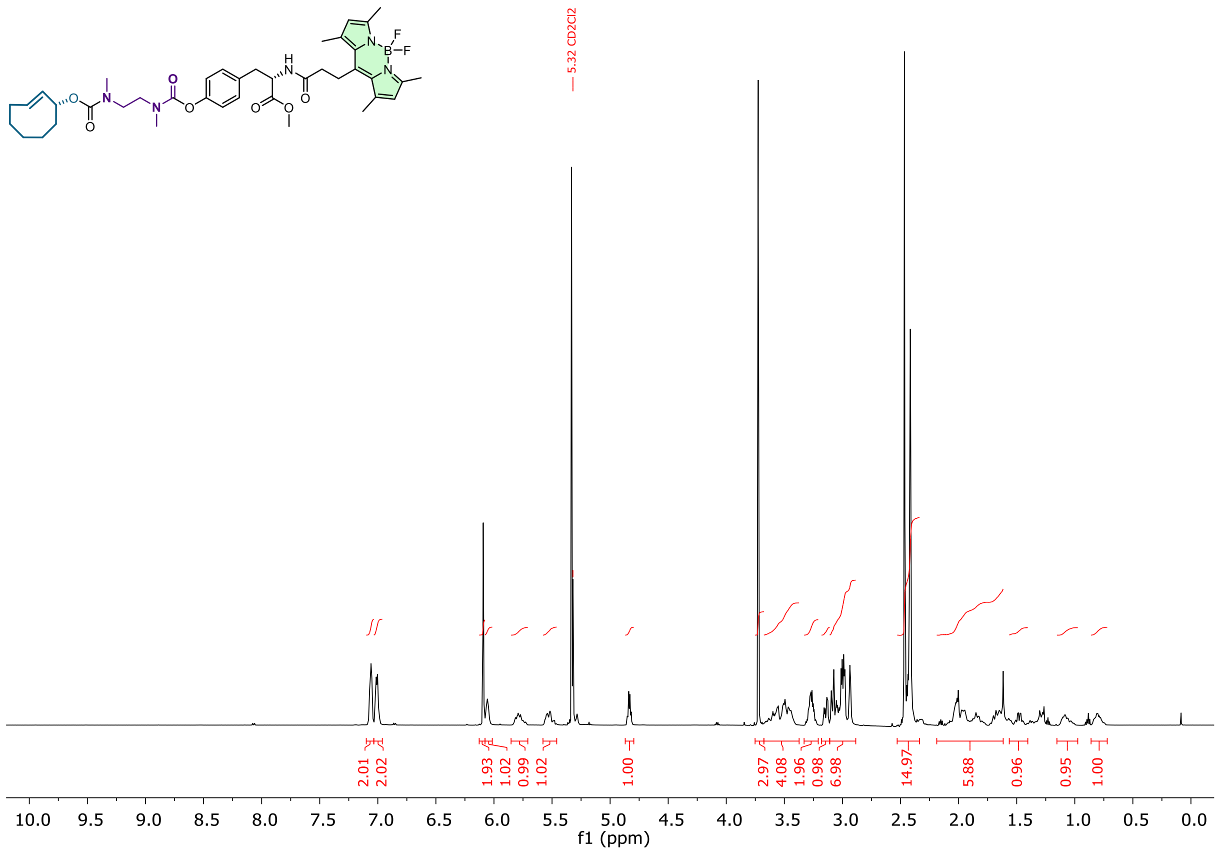

4, ^{13}C NMR

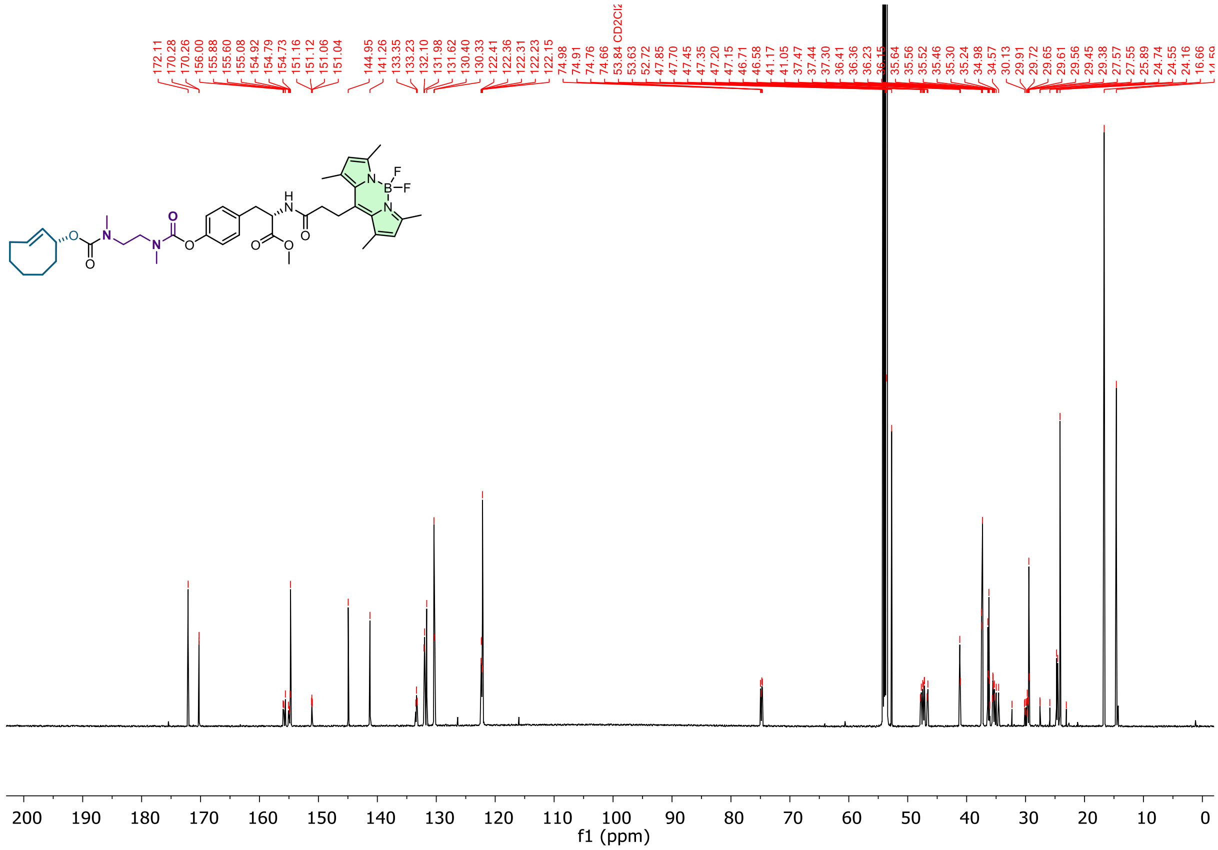

4, HPLC (acidic conditions)

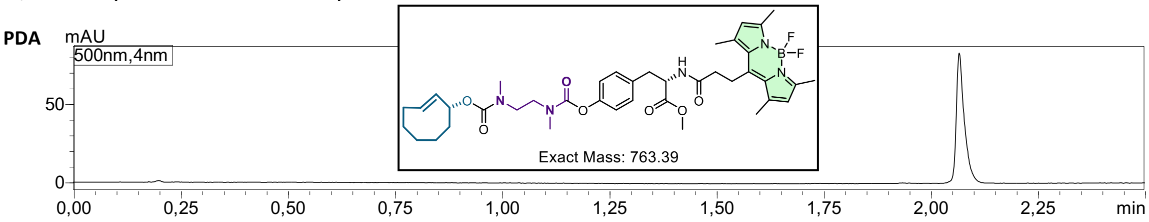

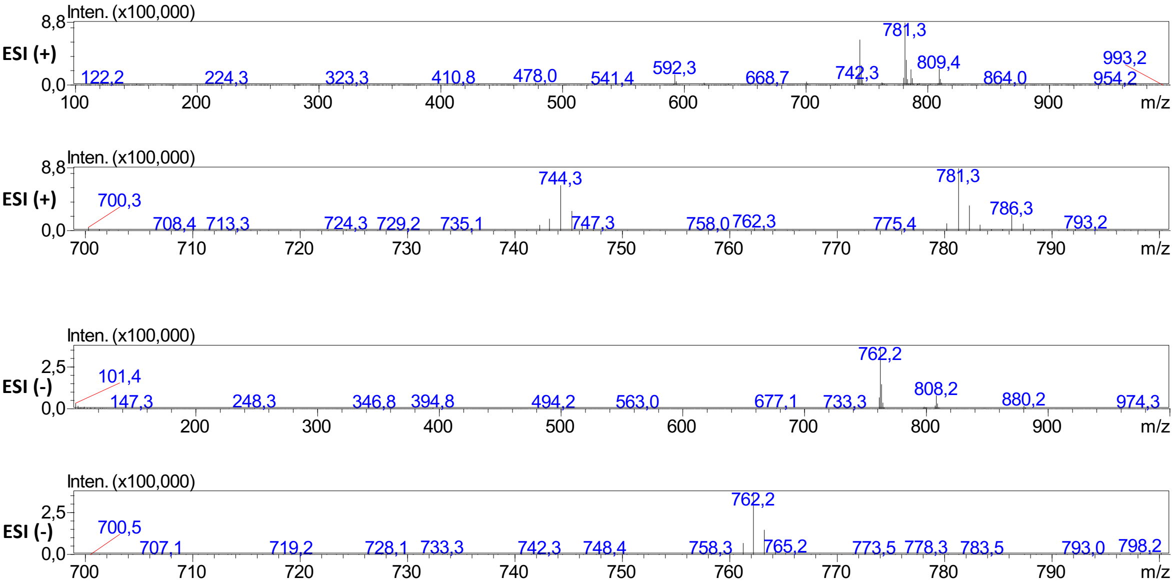

6, ^{1}H NMR

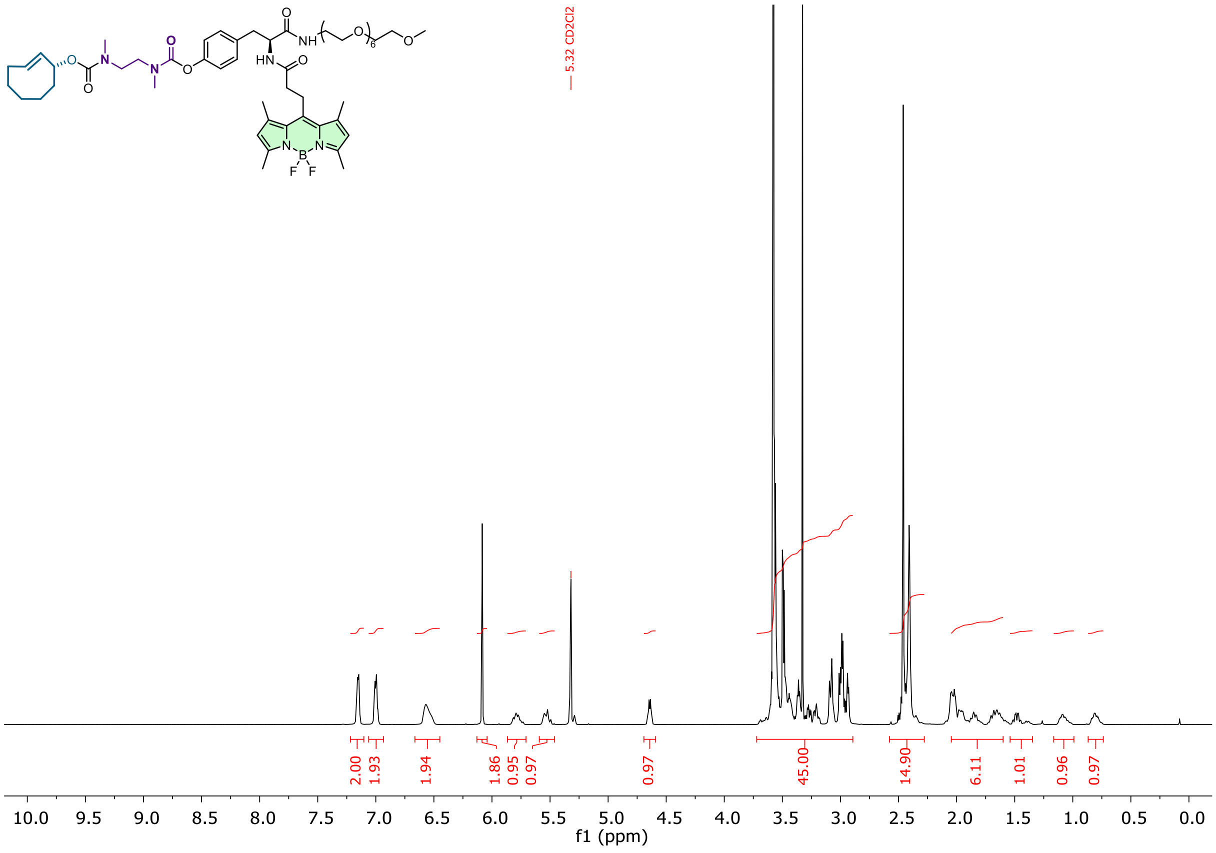

6, ^{13}C NMR

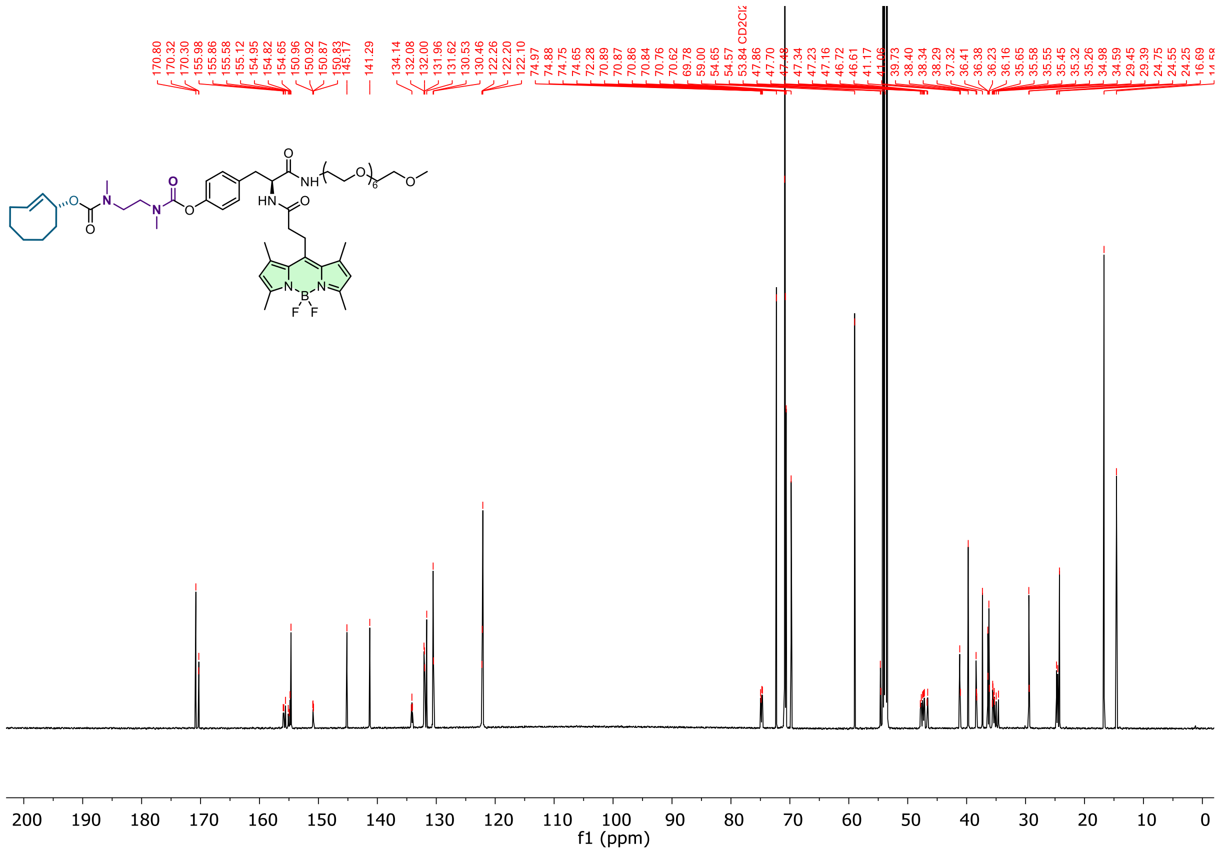

6, HPLC (acidic conditions)

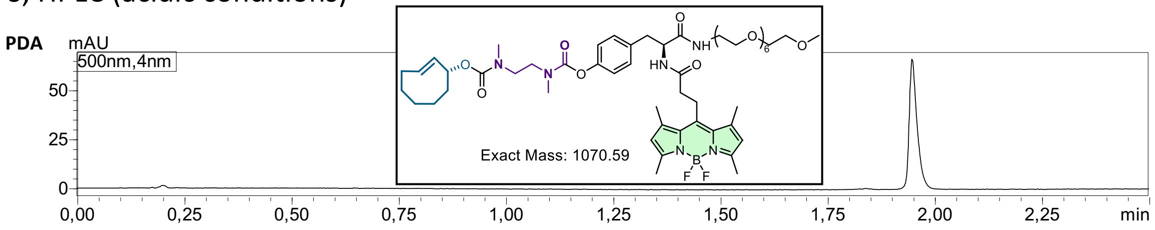

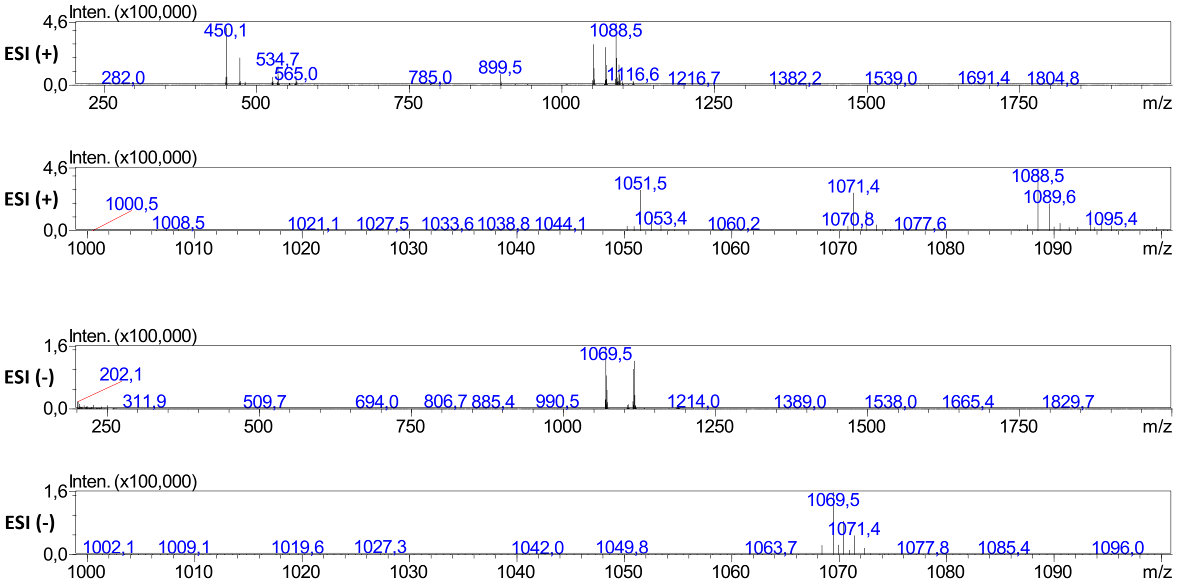

10, ^{1}H NMR

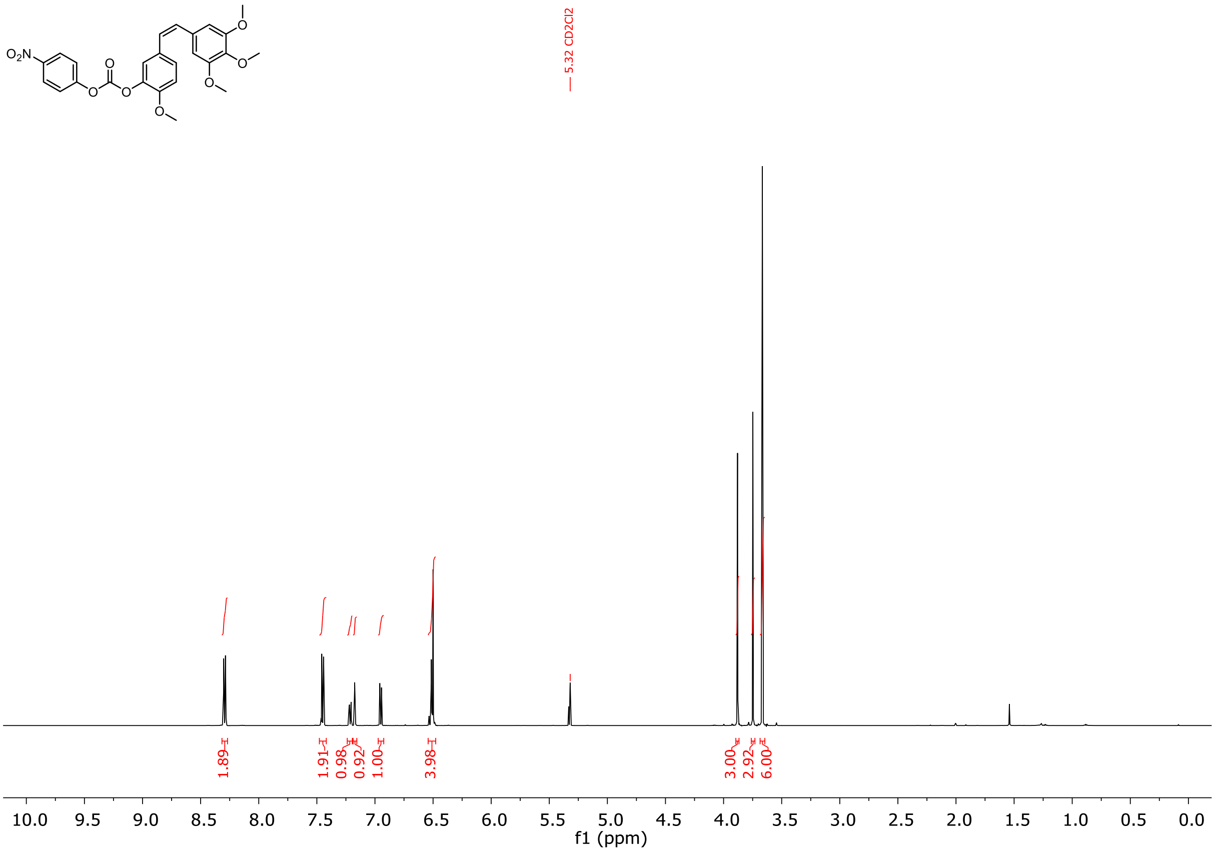

10, ^{13}C NMR

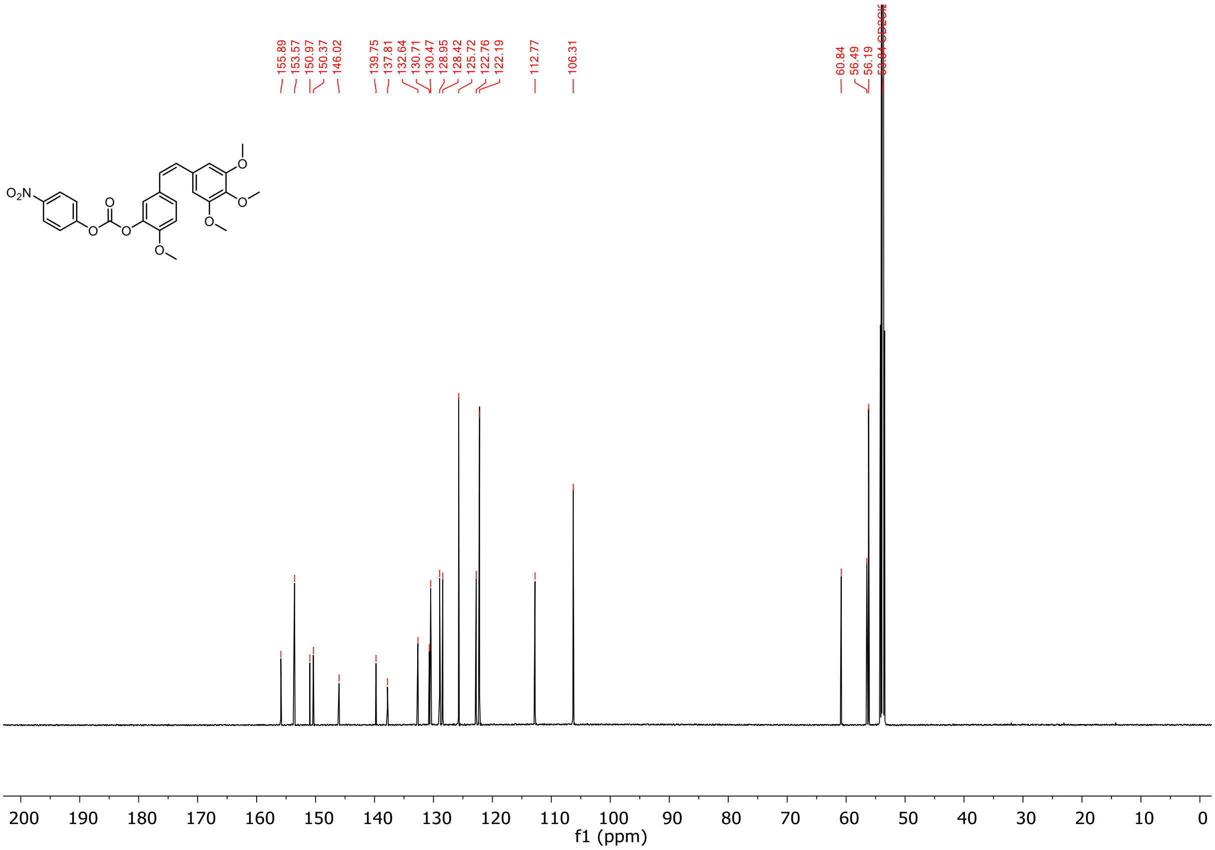

10, HPLC (acidic conditions)

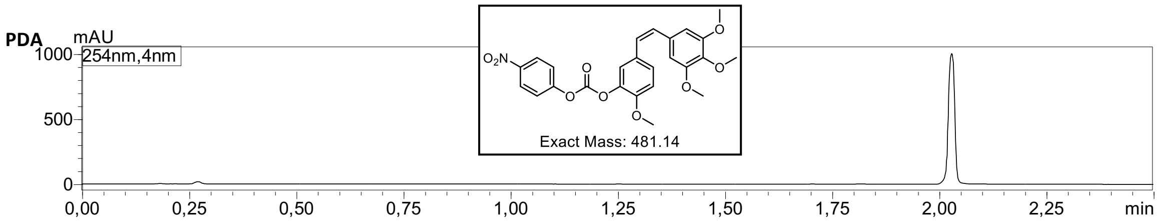

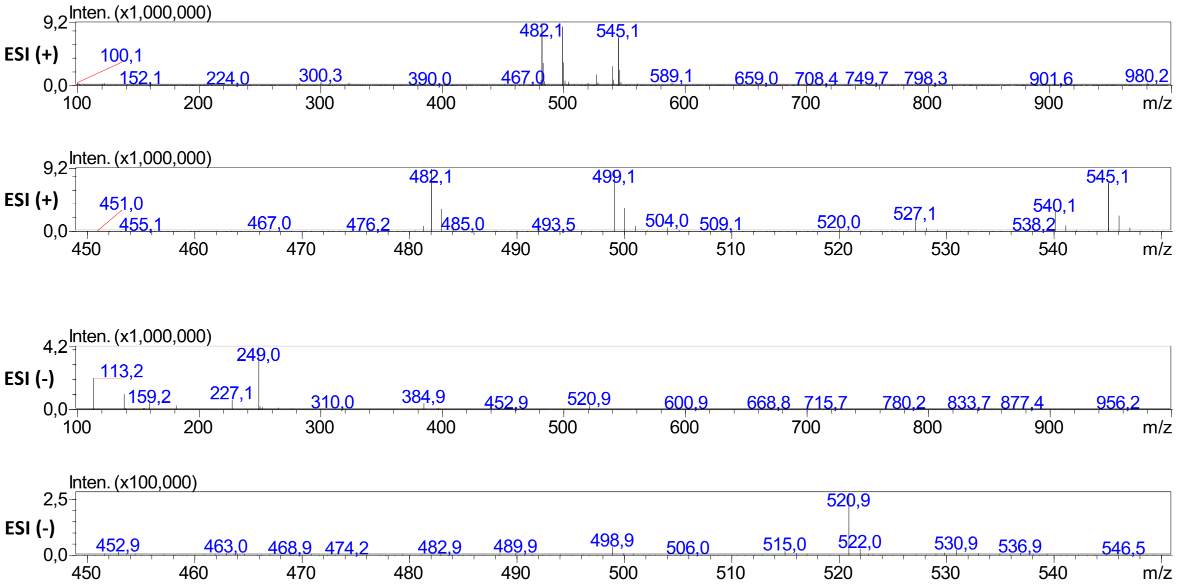

11, ^{1}H NMR

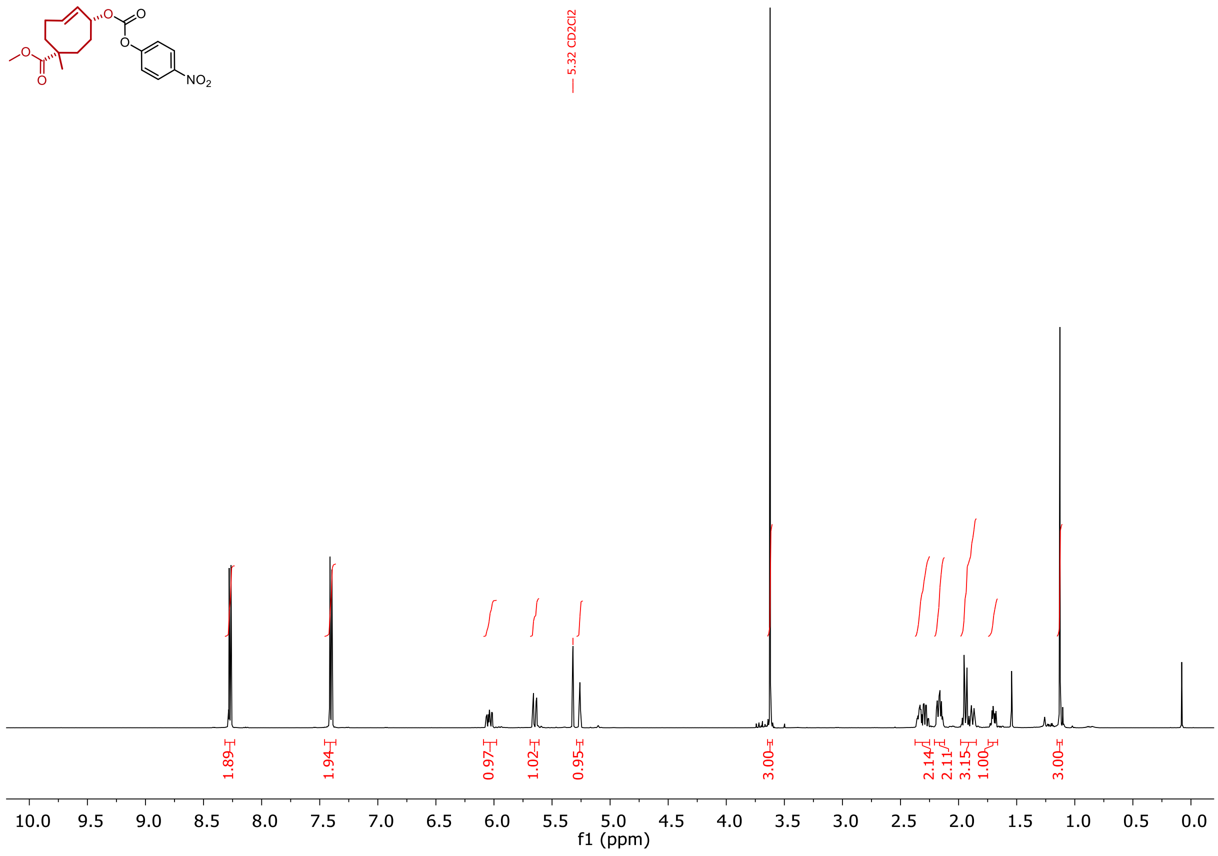

11, ^{13}C NMR

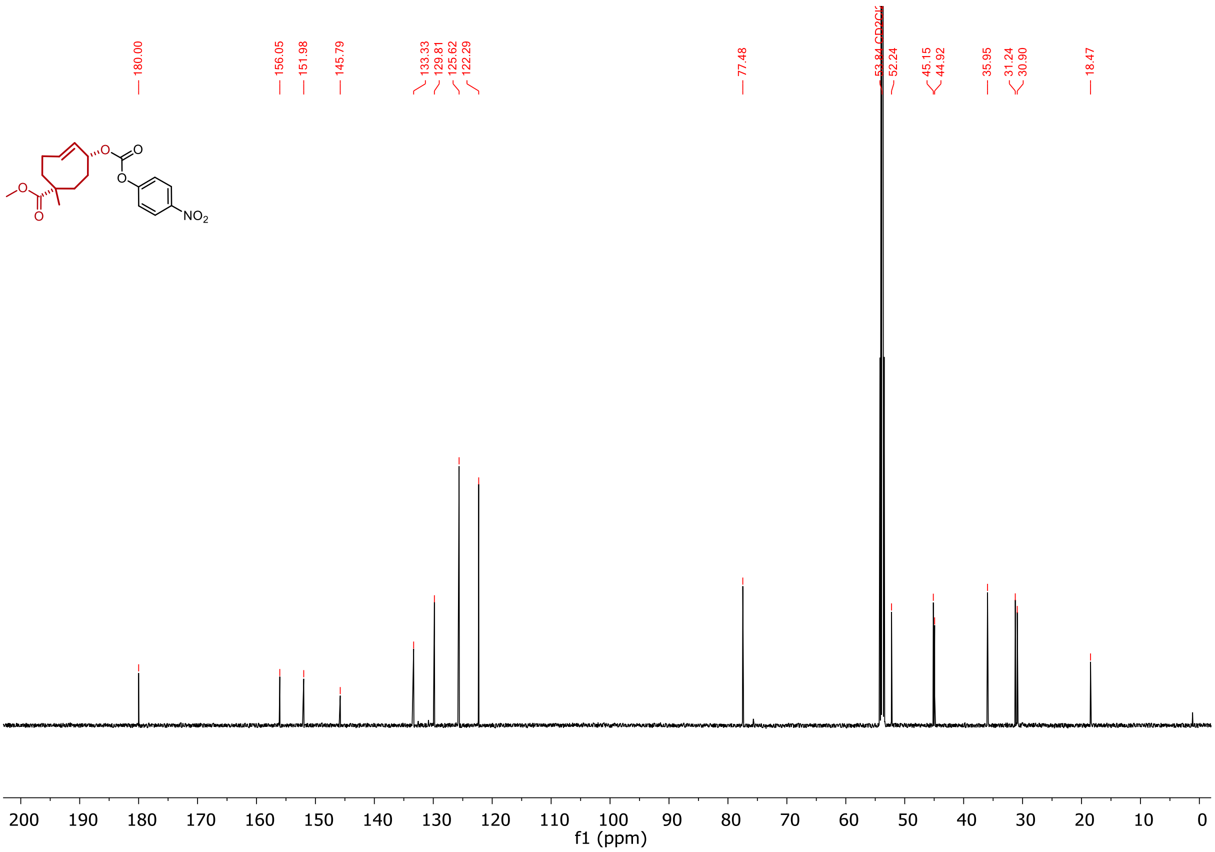

11, HPLC (acidic conditions)

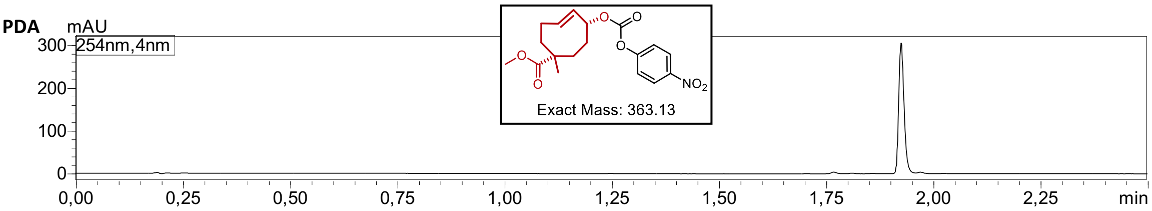

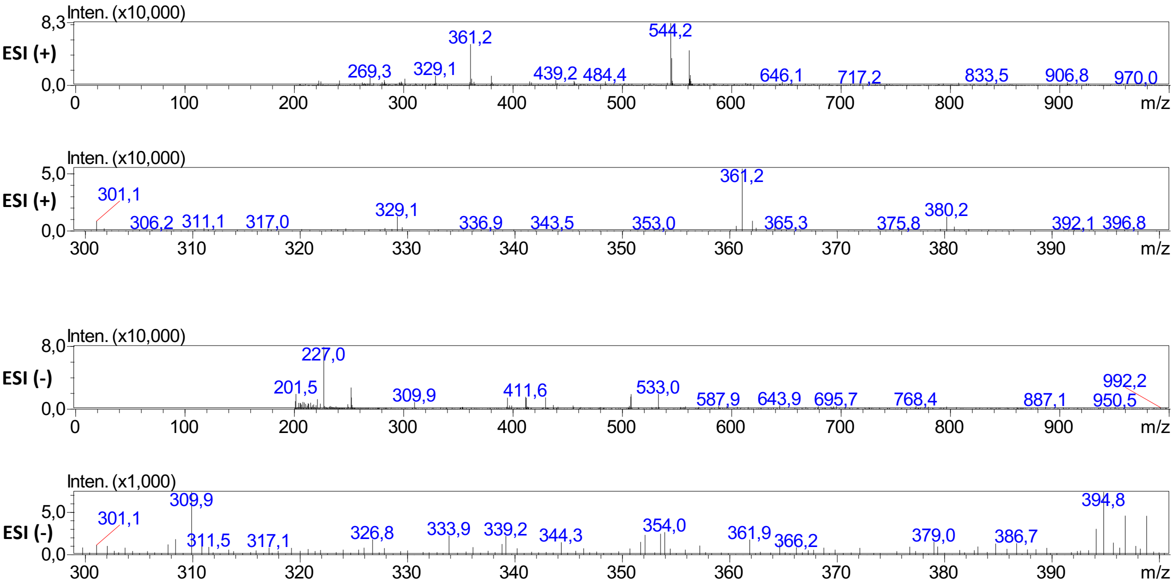

S3, ^{1}H NMR

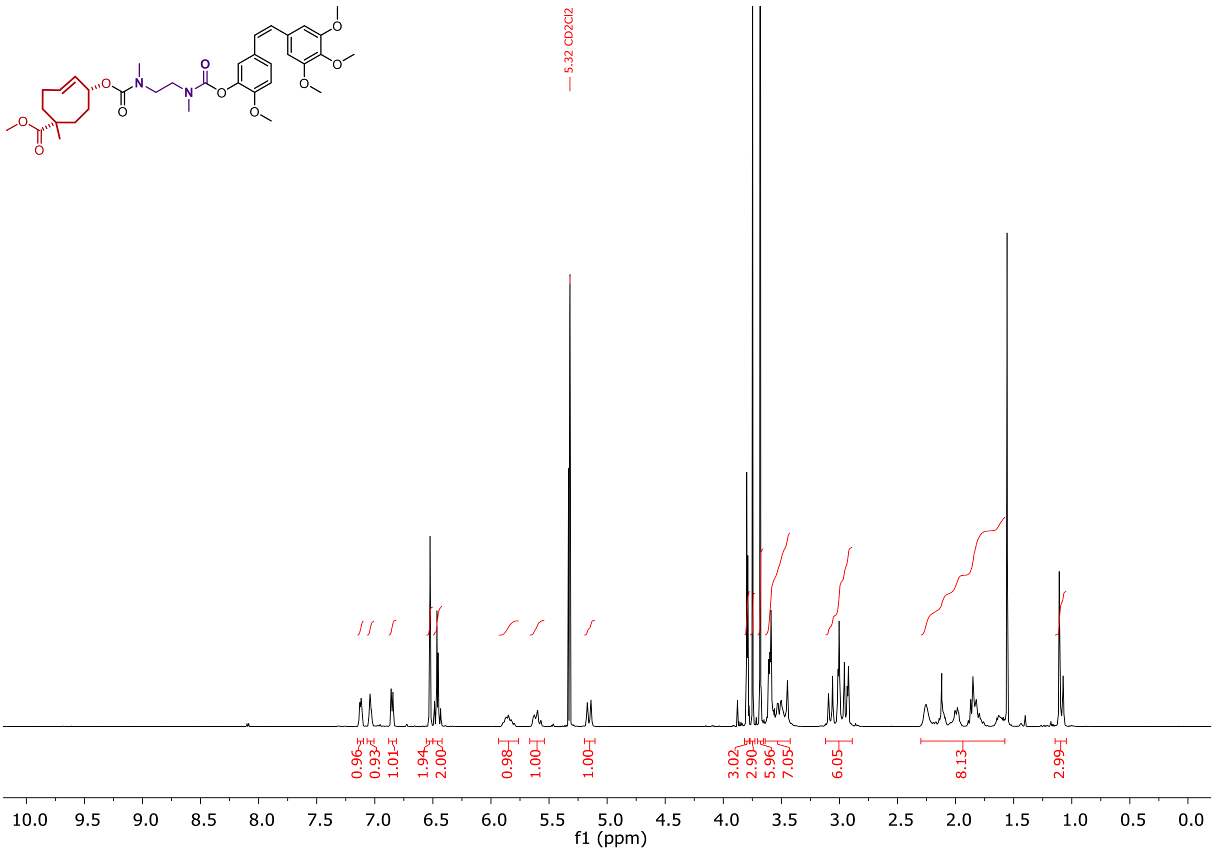

S3, ^{13}C NMR

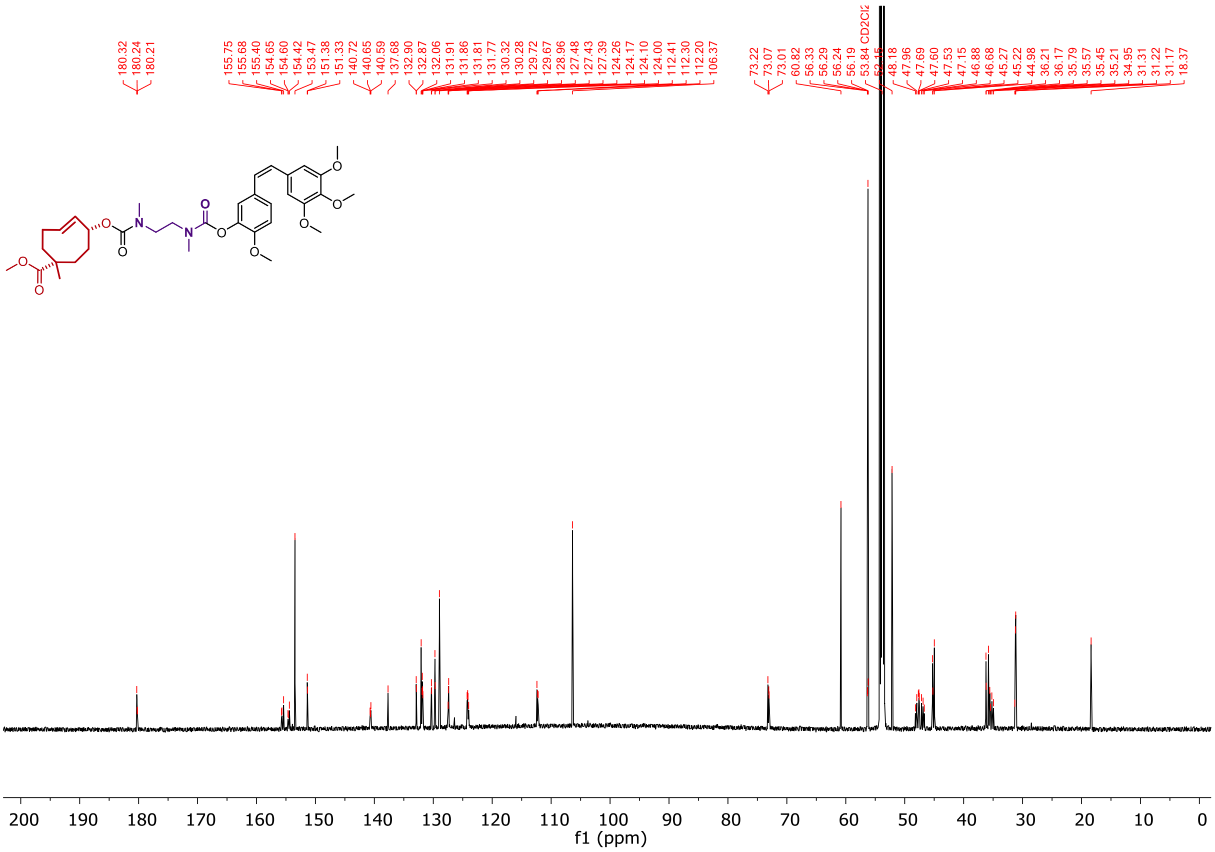

S3, HPLC (acidic conditions)

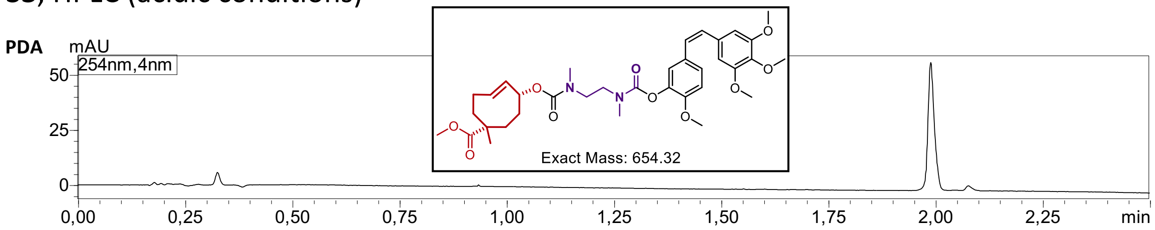

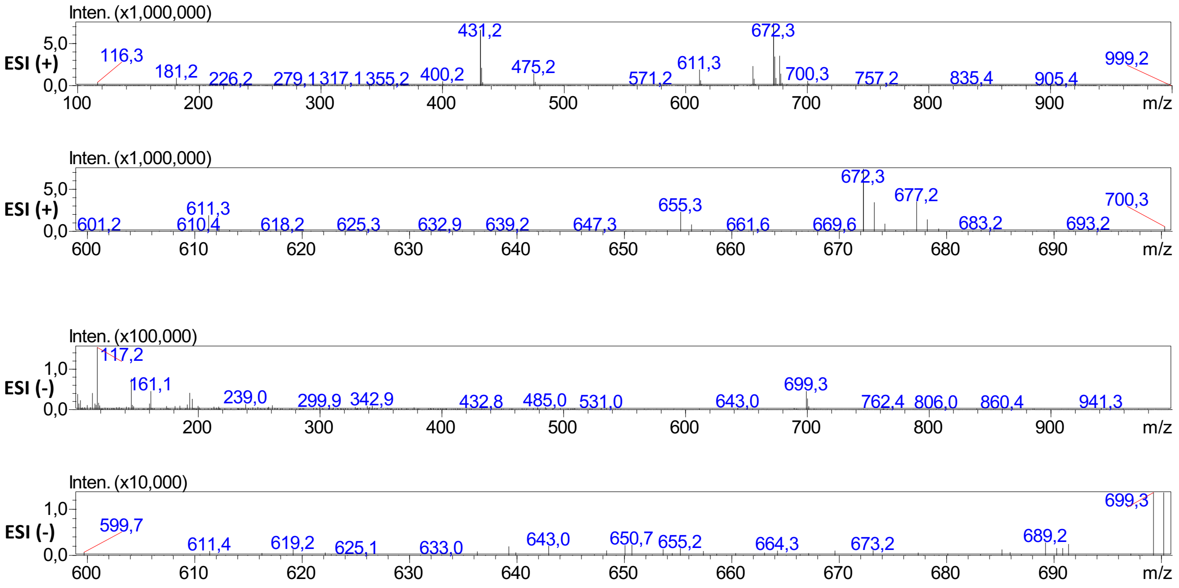

12, ¹H NMR

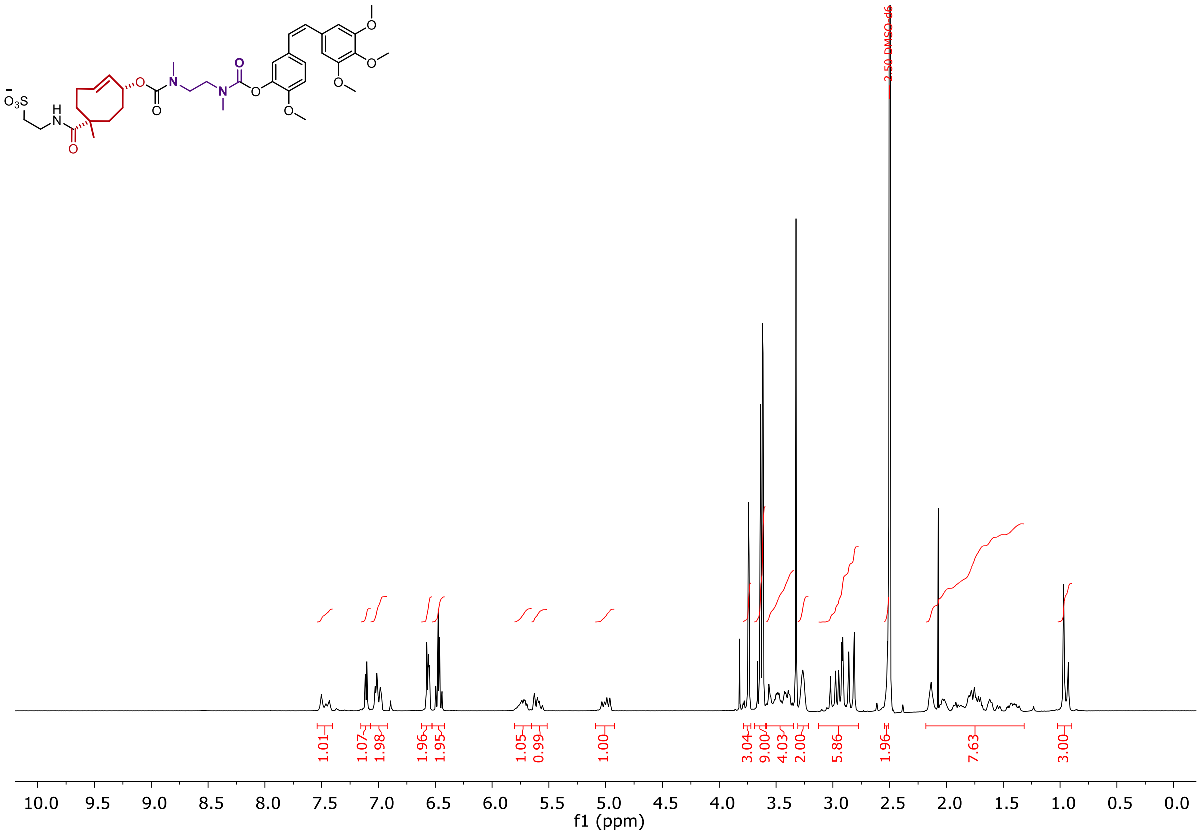

12, ^{13}C NMR

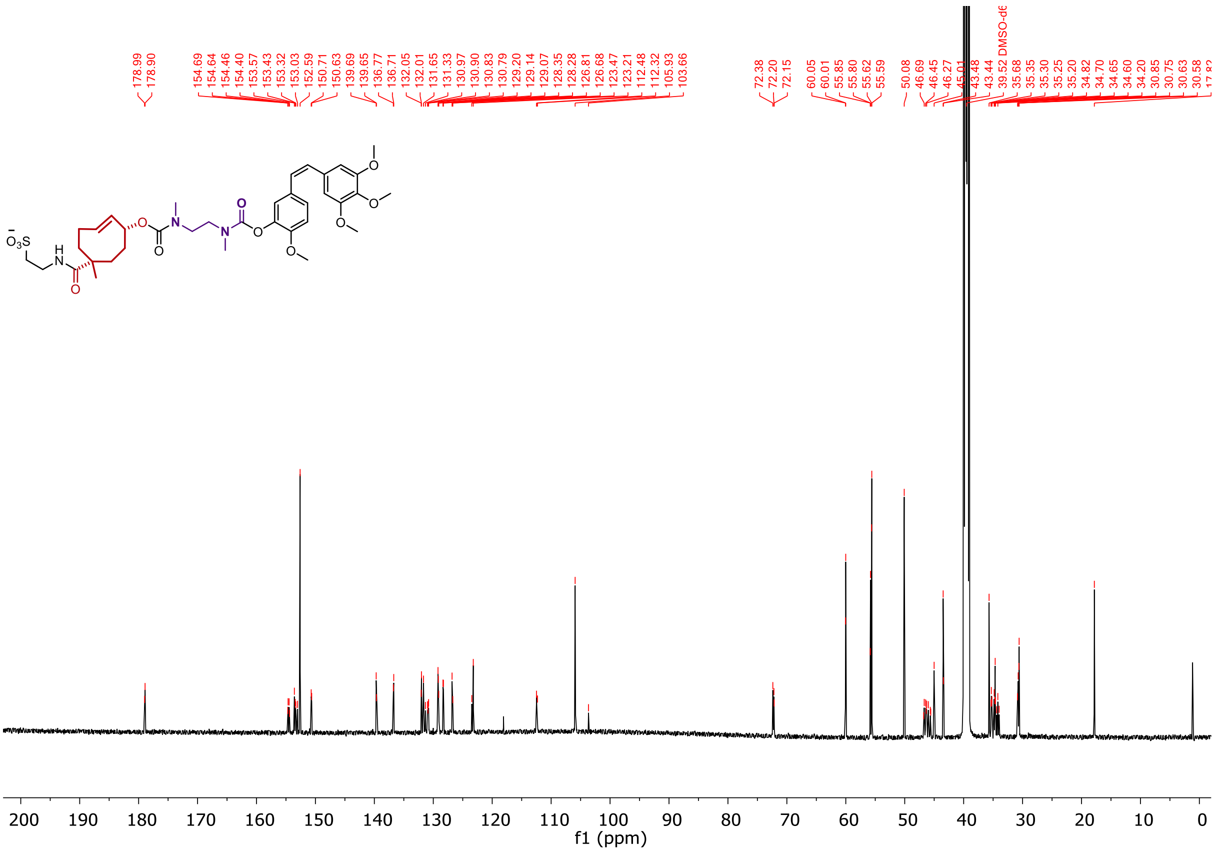

12, HPLC (basic conditions)

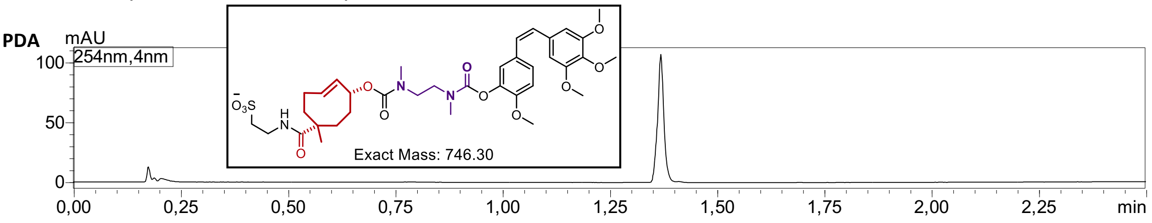

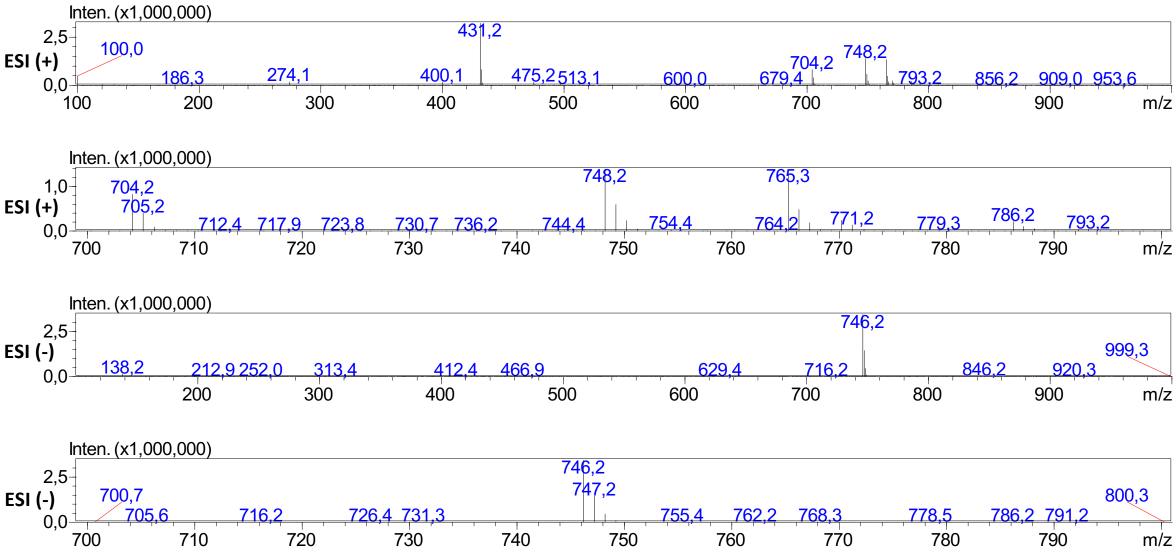

9 References

[1] D. Wang, J. Fan, X. Gao, B. Wang, S. Sun, X. Peng, *J. Org. Chem.* **2009**, *74*, 7675-7683.

[2] R. M. Versteegen, R. Rossin, W. ten Hoeve, H. M. Janssen, M. S. Robillard, *Angew. Chem. Int. Ed.* **2013**, *52*, 14112-14116.

[3] J. C. T. Carlson, H. Mikula, R. Weissleder, *J. Am. Chem. Soc.* **2018**, *140*, 3603-3612.

[4] A. J. C. Sarris, T. Hansen, M. A. R. de Geus, E. Maurits, W. Doelman, H. S. Overkleeft, J. D. C. Codee, D. V. Filippov, S. I. van Kasteren, *Chem. Eur. J.* **2018**, *24*, 18075-18081.

[5] R. Rossin, S. M. van Duijnhoven, W. Ten Hoeve, H. M. Janssen, L. H. Kleijn, F. J. Hoeben, R. M. Versteegen, M. S. Robillard, *Bioconjugate Chem.* **2016**, *27*, 1697-1706.

[6] G. Lukinavičius, L. Reymond, E. D'Este, A. Masharina, F. Göttfert, H. Ta, A. Güther, M. Fournier, S. Rizzo, H. Waldmann, C. Blaukopf, C. Sommer, D. W. Gerlich, H.-D. Arndt, S. W. Hell, K. Johnsson, *Nat. Methods* **2014**, *11*, 731-733.
